# Mitochondrial fission determines cisplatin sensitivity in tongue squamous cell carcinoma through the BRCA1–miR-593-5p–MFF axis

**DOI:** 10.18632/oncotarget.3659

**Published:** 2015-03-26

**Authors:** Song Fan, Bodu Liu, Lijuan Sun, Xiao-bin Lv, Zhaoyu Lin, Weixiong Chen, Weiliang Chen, Qionglan Tang, Youyuan Wang, Yuxiong Su, Shaowen Jin, Daming Zhang, Jianglong Zhong, Yilin Li, Bin Wen, Zhang Zhang, Pu Yang, Bin Zhou, Qixiang Liang, Xing Yu, Yinghua Zhu, Pengnan Hu, Junjun Chu, Wei Huang, Yuhuan Feng, Hongzhuang Peng, Qihong Huang, Erwei Song, Jinsong Li

**Affiliations:** ^1^ Guangdong Provincial Key Laboratory of Malignant Tumor Epigenetics and Gene Regulation, Sun Yat-Sen Memorial Hospital, Sun Yat-Sen University, Guangzhou, China; ^2^ Department of Oral & Maxillofacial Surgery, Sun Yat-sen Memorial Hospital, Sun Yat-sen University, Guangzhou, China; ^3^ Department of Breast Tumor Center, Sun Yat-sen Memorial Hospital, Sun Yat-sen University, Guangzhou, China; ^4^ Medical Research Center, Sun Yat-Sen Memorial Hospital, Sun Yat-sen University, Guangzhou, China; ^5^ Department of Pathology, Sun Yat-Sen Memorial Hospital, Sun Yat-Sen University, Guangzhou, China; ^6^ Division of Oral and Maxillofacial Surgery, Faculty of Dentistry, the University of Hong Kong, Hong Kong; ^7^ Department of Hepatobiliary Surgery, Sun Yat-Sen Memorial Hospital, Sun Yat-Sen University, Guangzhou, China; ^8^ Xaverian Brothers High School, Westwood, MA, USA; ^9^ Department of Pathology, The Affiliated Hospital of North Sichuan Medical College, Nanchong, China; ^10^ Department of Pathology, West China Hospital, Sichuan University, Chengdu, China; ^11^ State Key Laboratory of Oral Diseases, West China Hospital, Stomatology of Sichuan University, Chengdu, China; ^12^ The Wistar Institute, Philadelphia, PA, USA

**Keywords:** mitochondrial fission, cisplatin sensitivity, BRCA1, miR-593-5p, MFF

## Abstract

Cisplatin has been widely employed as a cornerstone chemotherapy treatment for a wide spectrum of solid neoplasms; increasing tumor responsiveness to cisplatin has been a topic of interest for the past 30 years. Strong evidence has indicated that mitochondrial fission participates in the regulation of apoptosis in many diseases; however, whether mitochondrial fission regulates cisplatin sensitivity remains poorly understood. Here, we show that MFF mediated mitochondrial fission and apoptosis in tongue squamous cell carcinoma (TSCC) cells after cisplatin treatment and that miR-593-5p was downregulated in this process. miR-593-5p attenuated mitochondrial fission and cisplatin sensitivity by targeting the 3′ untranslated region sequence of MFF and inhibiting its translation. In exploring the underlying mechanism of miR-593-5p downregulation, we observed that BRCA1 transactivated miR-593-5p expression and attenuated cisplatin sensitivity *in vitro*. The BRCA1-miR-593-5p-MFF axis also affected cisplatin sensitivity *in vivo*. Importantly, in a retrospective analysis of multiple centers, we further found that the BRCA1-miR-593-5p-MFF axis was significantly associated with cisplatin sensitivity and the survival of patients with TSCC. Together, our data reveal a model for mitochondrial fission regulation at the transcriptional and post-transcriptional levels; we also reveal a new pathway for BRCA1 in determining cisplatin sensitivity through the mitochondrial fission program.

## INTRODUCTION

Cisplatin was first approved by the Food and Drug Administration (FDA) in 1978 for the treatment of testicular and bladder cancer and has been largely employed as a cornerstone treatment in the fight against a wide spectrum of solid neoplasms, including (but not limited to) head and neck, colorectal, ovarian and lung cancers [1]. Cisplatin exerts anticancer effects via multiple mechanisms; however, its most prominent (and best understood) mode of action involves the generation of DNA lesions followed by the activation of the DNA damage response and the induction of mitochondrial apoptosis [1]. Although cisplatin often leads to initial therapeutic success, chemoresistance frequently develops and leads to therapeutic failure. The initial patient responsiveness to platinum-based therapies in oral squamous cell carcinoma (OSCC) is 80.6% [2]; however, more than 70% of patients eventually relapse because their tumors become resistant [3]. Intense research has investigated this phenomenon over the past 30 years, and several mechanisms have been described to be associated with the cisplatin-resistant phenotype of tumor cells, including decreased cellular drug accumulation, increased levels of glutathione, increased levels of DNA repair and increased anti-apoptotic activity [4]. Unfortunately, no substantive progress has been made in overcoming cisplatin resistance in a clinical setting due to the numerous resistance mechanisms that cancer cells have. Searching for other mechanisms through which cisplatin can exert its apoptotic effects may be the most practical avenue for achieving optimal effectiveness for this drug in a clinical setting.

Mitochondria have an important role in the initiation of apoptosis [5, 6]. In the intrinsic pathway, it is generally accepted that mitochondrial outer membrane permeabilization (MOMP), which leads to the release of pro-apoptotic proteins from the mitochondrial intermembrane space (IMS), is the most crucial event driving initiator caspase activation and apoptosis. However, recent evidence has revealed that certain cell types survive MOMP [7, 8]. For example, MOMP could be incomplete when some mitochondria fail to undergo MOMP following an apoptotic stimulus. Therefore, other mitochondrial activities may stimulate the intrinsic pathway. Interestingly, recent studies have revealed that an abnormal mitochondrial dynamic participates in the regulation of apoptosis [9]; this dynamic has been linked to a variety of diseases, such as skeletal muscle disorders [10, 11], Charcot-Marie-Tooth type 2A peripheral neuropathy [12], neurodegeneration [13], acute kidney injury [14] and myocardial infarction [15, 16]. Although the relationship between MOMP and mitochondrial fission during apoptosis in mammalian cells is unclear [17], mitochondrial fission appears to occur early in the apoptotic pathway or prior to MOMP [18] and can even be dissociated from MOMP [19]. Limited studies have shown that cisplatin can induce apoptosis in Hela cells [20] and ovarian cancer cells [21] through mitochondrial fission; nevertheless, no studies have indicated whether mitochondrial fission can predict cisplatin sensitivity in a clinical setting. In addition, whether mitochondrial fission participates in the cisplatin sensitivity of tongue squamous cell carcinoma (TSCC) should also be investigated.

DRP1 is a prominent dynamin-related GTPase in mammals that induces mitochondrial fission by generating the mechanical force that constricts (mechanistically pinches) mitochondria [22]. Multiple integral outer-membrane proteins (including FIS1, MID49/51 and MFF) work as DRP1 receptors to recruit DRP1 to mitochondria. However, there are conflicting results regarding whether inhibiting DRP1 enhances spontaneous apoptosis *in vitro* and *in vivo* in several cancer types, including colon, breast, lung and cervical cancers [23]. Additionally, in a previous study, no significant difference was found in cumulative survival between patients with high and low DRP1 levels in lung adenocarcinomas [24]. Consequently, the data suggest that DRP1 executes mitochondrial fission and apoptosis in a manner that is co-regulated with its pivotal receptor. However, the role of FIS1 and MID49/51 as outer-membrane proteins (tethers for DRP1) has recently been challenged [25, 26]. FIS1 overexpression affects neither mitochondria-associated DRP1 nor mitochondrial fission [27], whereas MiD49 recruits DRP1 to the mitochondrial outer membranes and promotes mitochondrial fusion rather than fission in vertebrates [28]. By contrast, other studies have clearly demonstrated that MFF penetrates into the mitochondrial outer membrane prior to DRP1 recruitment [29, 30], and DRP1 and MFF co-localization structures induce mitochondrial fission [31, 32]. However, no studies have indicated whether MFF affects cisplatin sensitivity through mitochondrial fission. In this study, we focused on MFF-dependent mitochondrial fission and revealed a novel mechanism of cisplatin sensitivity.

MiRNAs have been implicated in the regulation of numerous cellular processes. Some miRNAs have been found to regulate cisplatin sensitivity in cancer cells [33]. However, it is unknown whether miRNAs could regulate cisplatin sensitivity through the mitochondrial fission pathway. Interestingly, miRNAs have been reported to regulate mitochondrial fission by targeting DRP1 and FIS1 in mouse cardiomyocytes. Therefore, the role of miRNA in cancer cell mitochondrial fission requires further investigation.

The present study revealed that MFF regulates mitochondrial fission and cisplatin sensitivity in TSCC cells. miR-593-5p represses MFF expression by targeting the MFF mRNA 3′-UTR. BRCA1 is generally thought to regulate cisplatin sensitivity through DNA damage repair; however, our *in vivo* and *in vitro* experiments showed that BRCA1 transactivates miR-593-5p expression and inhibits MFF expression through transcriptionally targeting miR-593-5p, consequently regulating mitochondrial fission and cisplatin sensitivity. Our results reveal a model for the BRCA1-miR-593-5p–MFF axis in mediating mitochondrial fission in cancer cells. More importantly, the BRCA1-miR-593-5p–MFF axis is related to cisplatin sensitivity and the survival of TSCC patients; this discovery may provide novel regulatory factors for enhancing cisplatin sensitivity in a clinical setting.

## RESULTS

### MFF regulates mitochondrial fission and cisplatin sensitivity

Cisplatin can induce apoptosis by initiating a mitochondrial fission pathway [20, 21]. However, the underlying mechanism of this effect remains elusive. To study the mechanism through which mitochondrial fission regulates cisplatin sensitivity in TSCC, we first measured morphological changes in TSCC mitochondria after cisplatin stimulation(Supplementary Figure S1). We observed that mitochondrial fission increased in TSCC cells (Supplementary Figure S1B) and that increased levels of cytochrome c(CYTO c) were released from the intermembrane space of the mitochondria to the cytosol (Supplementary Figure S1C) after cisplatin treatment. These results indicate that mitochondrial fission participates in the apoptosis of TSCC cells after cisplatin treatment.

Growing evidence has demonstrated that MFF primarily penetrates the mitochondrial outer membrane and recruits DRP1 to initiate mitochondrial fission and cell apoptosis [29-32]; however, little is known regarding the relationship between MFF and cisplatin sensitivity. Therefore, we tested whether cisplatin affects mitochondrial fission and apoptosis in TSCC cells via MFF-dependent machinery.

**Figure 1:**
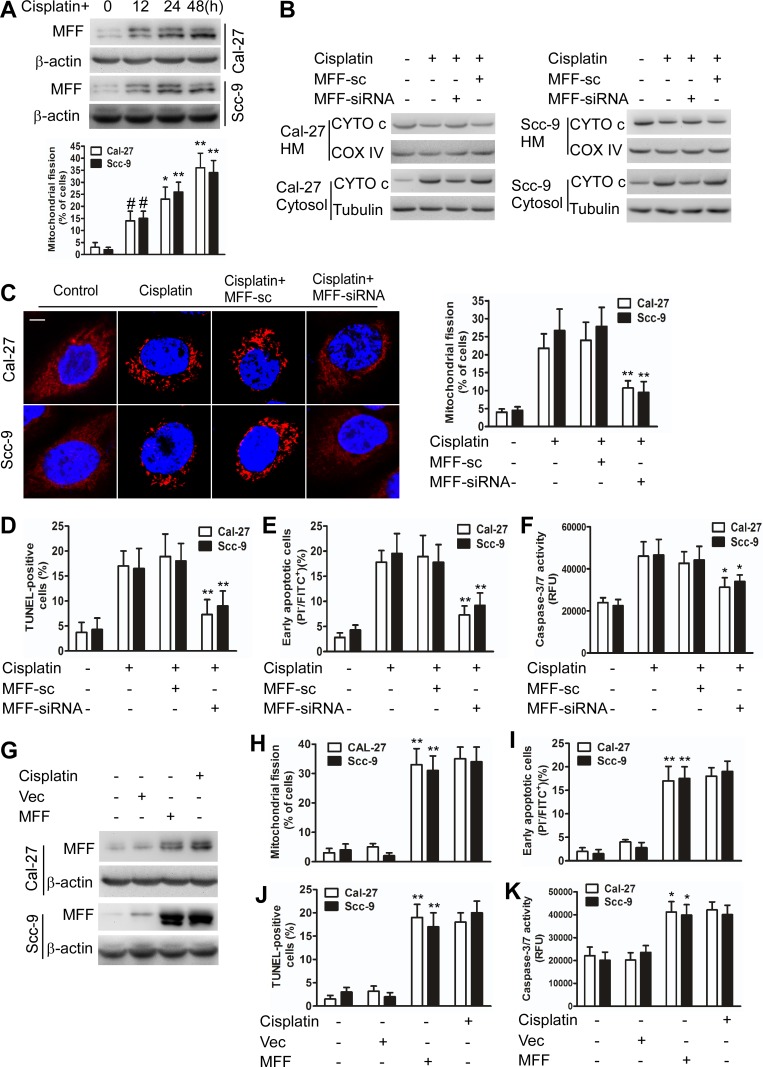
MFF regulates mitochondrial fission and apoptosis in TSCC cells after cisplatin treatment **A**, Cisplatin induces mitochondrial fission with elevated MFF protein levels in Cal-27 and Scc-9 cells. Upper panel: MFF levels were analyzed via immunoblotting after cisplatin treatment. Lower panel: the quantification of cells with mitochondrial fission. ^#^P < 0.05 versus no cisplatin treatment; *P < 0.01 versus no cisplatin treatment; **P < 0.001 versus no cisplatin treatment. **B**, Cytochrome c (CYTO c) distribution in mitochondria-enriched heavy membranes (HM) or the cytosol as detected via immunoblotting. **C**, **D**, **E** and **F**, Knockdown of MFF attenuated cisplatin-induced mitochondrial fission and apoptosis in Cal-27 and Scc-9 cells. Mitochondrial fission was detected via staining with MitoTracker Red. Scale bar equals 3 μm. Cell apoptosis was detected using TUNEL, flow cytometry, and caspase-3/7 activity assays. *P < 0.01 versus cisplatin alone; **P< 0.001 versus cisplatin alone. **G**, Cal-27 and Scc-9 cells transiently transfected with MFF expressing plasmids for 24 h were analyzed for MFF levels via immunoblotting. **H**, **I**, **J** and **K**, Mitochondrial fission and apoptosis were detected via staining with MitoTracker Red, flow cytometry, TUNEL, and caspase-3/7 activity assays. *P < 0.01 versus no cisplatin treatment; **P < 0.001 versus no cisplatin treatment.

Cisplatin induced mitochondrial fission with elevated MFF protein levels (Figure 1A), but not elevated mRNA levels (Supplementary Figure S2A). Immunofluorescence microscopy revealed that MFF exhibited punctate localization in mitochondria and that mitochondria fragmentation occurred upon cisplatin treatment of TSCC cells (Supplementary Figure S2B). MFF knockdown attenuated the MFF protein upregulation (Supplementary Figure S2C) and partially inhibited the release of cytochrome c in the intermembrane space of mitochondria (Figure 1B) of cisplatin-treated cells. Cisplatin induced an alteration in the expression of FIS1, DRP1, MFN1, MFN2 and optic atrophy type I (OPA1); this alteration was not affected by MFF siRNA (Supplementary Figure S2C). Consequently, mitochondrial fission (Figure 1C) and the apoptosis of TSCC cells (Figure 1D-F) were attenuated by MFF siRNA. By contrast, enforced MFF expression led to mitochondrial fission and apoptosis (Figure 1G-K). These data suggest that MFF regulates mitochondrial fission and cisplatin sensitivity in TSCC cells.

### miR-593-5p regulates mitochondrial fission and cisplatin sensitivity through MFF

**Figure 2:**
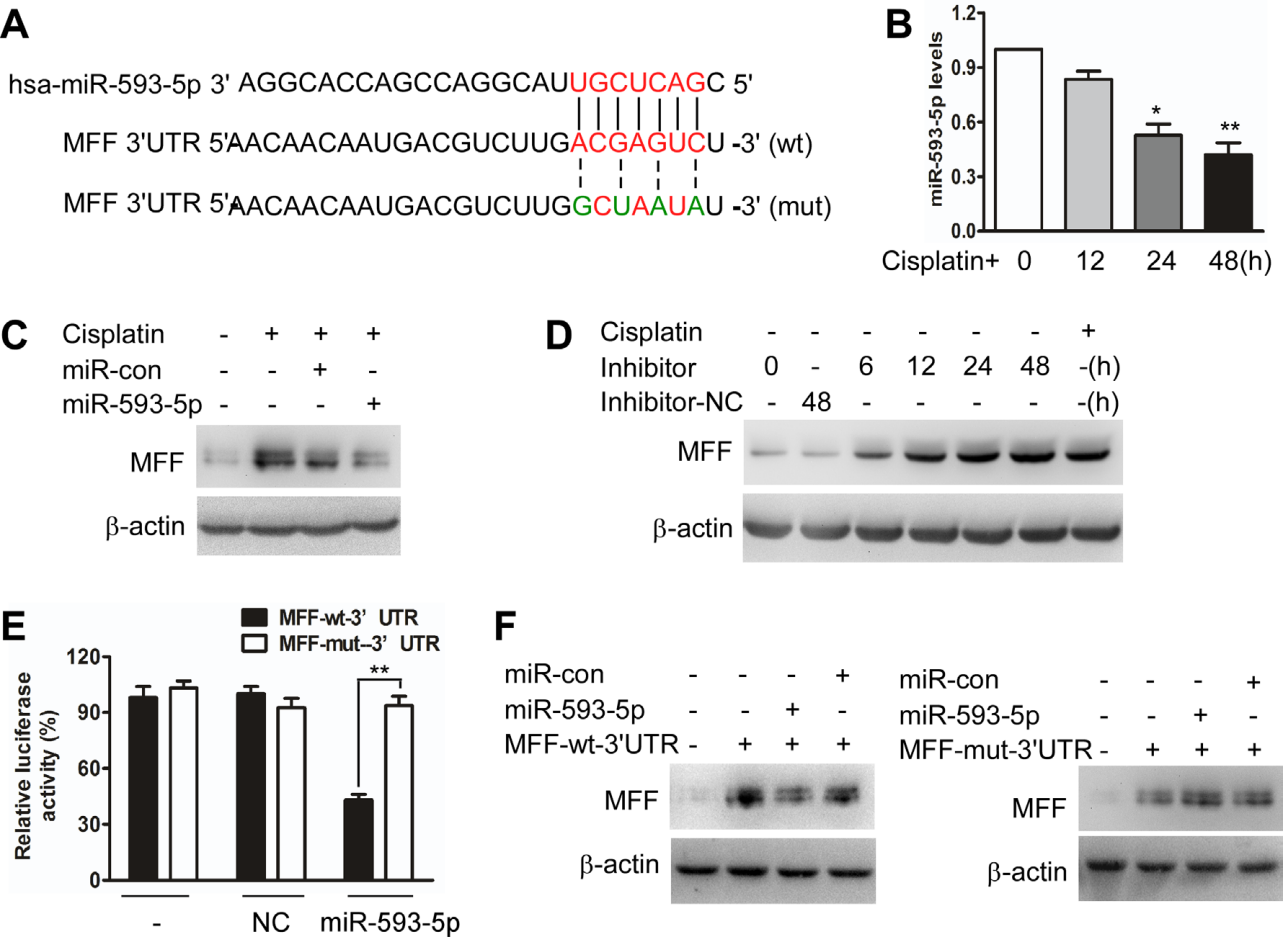
miR-593-5p targets the 3′UTR of MFF **A**, Analysis of miR-593-5p targeting sites in MFF 3′UTR via regrna2 (http://regrna2.mbc.nctu.edu.tw) and the construction of MFF 3′UTR mutants(mut). **B**, qRT-PCR was performed to analyze miR-593-5p levels under cisplatin treatment in Cal-27 cells. *P < 0.01 versus no cisplatin treatment; **P < 0.001 versus no cisplatin treatment. **C**, Forced expression of miR-593-5p attenuated the cisplatin-induced increase in MFF protein levels. Cal-27 cells were transfected with miR-593-5p mimics or miR-593-5p control (miR-con). After 24h of transfection, the cells were exposed to cisplatin and harvested 24 h after treatment for MFF analysis via immunoblotting. **D**, Knockdown of miR-593-5p upregulated MFF levels. Cal-27 cells were transfected with miR-593-5p inhibitors or negative control(inhibitors-NC). After 24 h of transfection, the cells were harvested for MFF analysis via immunoblotting. **E**, A luciferase assay was performed in Cal-27 cells that were co-transfected with miR-593-5p mimics and reporter vectors carrying MFF 3′UTR with wild type (MFF-wt-3′UTR) versus mutated (MFF-mut-3′UTR) miR-593-5p response element. **P < 0.001. **F**, miR-593-5p suppressed MFF expression with wild type but not mutated 3′UTR. Cal-27 cells were co-transfected with miR-593-5p mimics or control (miR-con) along with pcDNA3.1 cloned with a wild-type (MFF-wt-3′UTR) or mutatated (MFF-mut-3′UTR) MFF expression cassette at miR-593-5p response element. MFF levels were detected using immunoblotting.

To elucidate the molecular mechanisms by which MFF protein levels, but not mRNA levels, are upregulated, we tested whether miRNAs control MFF expression. We analyzed potential targets using a bioinformatics program (http://regrna2.mbc.nctu.edu.tw). We found that the 3′untranslated region (3′UTRs) of MFF had binding sites for miR-593-5p (Figure 2A). We first found that miR-593-5p was downregulated after cisplatin exposure in Cal-27 cells (Figure 2B). The enforced expression of miR-593-5p increased miR-593-5p levels (Supplementary Figure S3A) and attenuated the increase in MFF protein levels after cisplatin exposure (Figure 2C). By contrast, MFF mRNA levels were not altered by miR-593-5p under cisplatin conditions (Supplementary Figure S3B). DRP1 and FIS1 protein levels were also unchanged (Supplementary Figure S3C). The knockdown of endogenous miR-593-5p resulted in elevated MFF levels (Figure 2D). To determine whether MFF is a direct target of miR-593-5p, we tested the effect of miR-593-5p on MFF translation. The introduction of mutations of MFF-3′UTRs that disrupt base pairing between miR-593-5p and MFF rescued luciferase expression (Figure 2E) and MFF protein expression (Figure 2F). Together, these results suggest that miR-593-5p directly targets MFF-3′UTRs.

**Figure 3:**
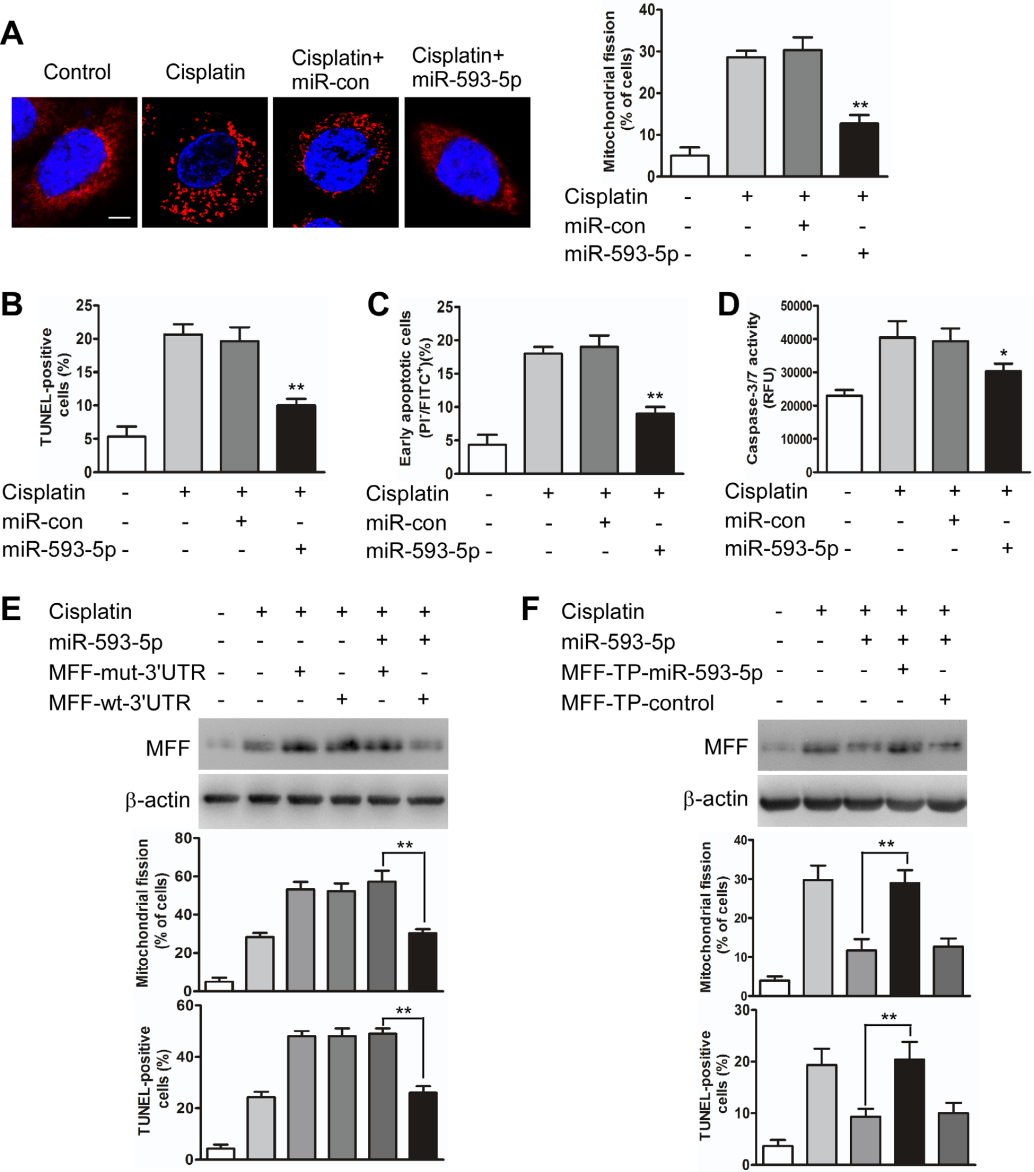
miR-593-5p regulates mitochondrial fission and apoptosis in Cal-27 cells **A**, miR-593-5p prevents mitochondrial fission. Cal-27 cells were transfected with miR-593-5p mimics or miR-593-5p control (miR-con). After 24 h of transfection, the cells were treated with cisplatin for 24 h followed by staining with MitoTracker Red. Left panel: mitochondria were visualized via staining with mitoTracker Red. The scale bar equals 3 μm. Right panel: quantification of cells with mitochondrial fission. **P < 0.001 versus cisplatin alone. **B**, **C** and **D**, Apoptosis was detected via TUNEL assay, flow cytometry and caspase-3/7 assay. *P < 0.01 versus cisplatin alone; **P< 0.001 versus cisplatin alone. **E**, miR-593-5p attenuated MFF levels, mitochondrial fission and apoptosis in the presence of MFF with wild type 3′UTR (MFF-wt-3′UTR) but not its mutated 3′UTR (MFF-mut-3′UTR). Cal-27 cells were transfected with miR-593-5p mimics along with pcDNA3.1 plasmid carrying a wild-type (MFF-wt-3′UTR) or mutatated (MFF-mut-3′UTR) MFF expression cassette at miR-593-5p response element. MFF levels were analyzed via immunoblotting (upper panel). Mitochondrial fission and apoptosis were detected via staining with MitoTracker Red and TUNEL. **P< 0.001. **F**, MFF target protector reduces the inhibitory effect of miR-593-5p on mitochondrial fission and apoptosis. Cal-27 cells were transfected with miR-593-5p mimics, along with the target protector (MFF-TP-miR-593-5p) or the control (MFF-TP-control). MFF was analyzed via immunoblotting (upper panel). Mitochondrial fission and apoptosis were detected via staining with MitoTracker Red and TUNEL. **P < 0.001.

Then, we tested whether MFF is a downstream target of miR-593-5p during the regulation of mitochondrial fission and cisplatin sensitivity. The enforced expression of miR-593-5p inhibited mitochondrial fission (Figure 3A) and apoptosis (Figure 3B-D) after cisplatin exposure in Cal-27 cells. However, this inhibitory effect was significantly abolished when Cal-27 cells were transfected with miR-593-5p mimics along with pcDNA3.1 plasmid cloned with MFF expression cassette containing mutant 3′UTR (MFF-mut-3′UTR) at miR-593-5p response element (Figure 3E, Supplementary Figure S3D). Target protector technology [34], which disrupts the interaction between miRNA-mRNA pairs, has been used to identify the specific recognition sequence of a miRNA to a target region. We employed an MFF target protector (TP) and found that the inhibitory effect of miR-593-5p on mitochondrial fission and apoptosis was reduced in the presence of the MFF target protector (Figure 3F, Supplementary Figure S3E). Furthermore, we attempted to determine whether the regulation of miR-593-5p on MFF was cell type-specific and observed that miR-593-5p levels were also decreased in Scc-9 cells after cisplatin exposure (Supplementary Figure S4A). Exogenous miR-593-5p expression concomitantly increased miR-593-5p levels(Supplementary Figure S4B) and attenuated MFF protein levels(Supplementary Figure S4C) without affecting MFF mRNA levels(Supplementary Figure S4D). Meanwhile, mitochondrial fission and cell apoptosis were also attenuated by exogenous miR-593-5p expression in Scc-9 cells(Supplementary Figure S4E and S4F). These data suggest that miR-593-5p functionally controls mitochondrial fission and cisplatin sensitivity through its MFF downstream target in TSCC cells.

### BRCA1 transactivates miR-593-5p and influences mitochondrial fission and cisplatin sensitivity through miR-593-5p and MFF

We investigated how miR-593-5p expression could be downregulated under cisplatin stress. miRNA expression has been reported to be regulated at the transcriptional level under physiological and pathological conditions [35, 36]. We analyzed the 5-kb region upstream of miR-593-5p and observed that it contains ten possible binding sites (BS) for the transcription factor BRCA1 (jaspar.genereg.net; Supplementary Figure S5A). This result led us to investigate whether BRCA1 is involved in the regulation of miR-593-5p expression.

**Figure 4:**
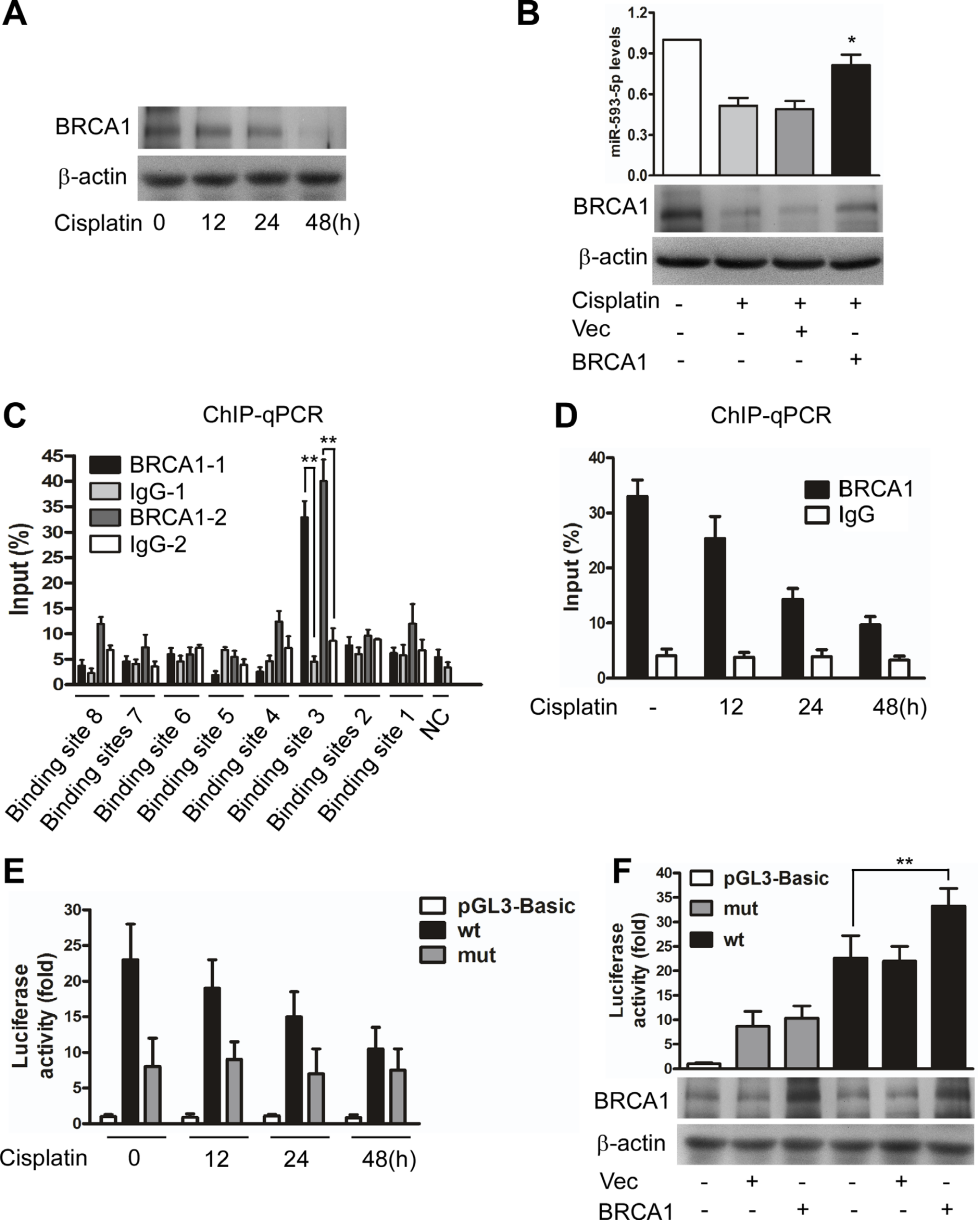
BRCA1 transactivates miR-593-5p **A**, BRCA1 was analyzed using immunoblotting in Cal-27 cells under cisplatin treatment. **B**, BRCA1 attenuated the cisplatin-induced decrease of miR-593-5p. Cal-27 cells were transiently transfected with BRCA1 expressing plasmids or empty vector (Vec) and then treated with cisplatin for 24h. miR-593-5p were detected using qRT-PCR (upper panel), whereas BRCA1 was analyzed using immunoblotting (lower panel). *P< 0.01 versus cisplatin alone. **C**, ChIP-qPCR analysis of BRCA1 binding to the promoter of miR-593-5p in the BS3 region. **P< 0.001. **D**, ChIP-qPCR analysis of the association levels of BRCA1 with the miR-593-5p promoter in the BS3 region under cisplatin treatment. **E**, A luciferase assay indicated that cisplatin induced a reduction of miR-593-5p promoter activity in the BS3 region. Cal-27 cells were transfected with the wild-type promoter (wt) in the BS3 or empty vector (pGL3-Basic). **F**, A luciferase assay indicated that BRCA1 activated miR-593-5p promoter activity in the BS3 region. Cal-27 cells transiently transfected with BRCA1 expressing plasmids or empty vector (Vec) were treated with the wild-type promoter (wt) or a promoter with mutations in the BS3 (mut). **P< 0.001.

First, we observed that BRCA1 was downregulated under cisplatin stress in Cal-27 cells (Figure 4A). BRCA1 siRNA (Supplementary Figure S5B) or the enforced expression of BRCA1 (Supplementary Figure S5C) downregulated or upregulated miR-593-5p expression, respectively, and expressing exogenous BRCA1 attenuated the cisplatin-induced downregulation of miR-593-5p (Figure 4B). Furthermore, a chromatin immunoprecipitation (ChIP) quantitative PCR (qPCR) assay revealed that BRCA1 bound to binding site 3 (BS3) but not to the other binding sites under physiological conditions (Figure 4C). Cisplatin treatment led to a reduced association of BRCA1 with the miR-593-5p promoter in the BS3 region (Figure 4D); the luciferase reporter assay also demonstrated reduced miR-593-5p promoter activity after cisplatin exposure (Figure 4E). BRCA1 overexpression increased miR-593-5p promoter activity; this enhancement was reversed through mutations introduced into the BS3 region (Figure 4F). miR-593-5p is located within the intron of the SND1 gene, but SND1 mRNA levels were not substantially altered after cisplatin treatment (Supplementary Figure S5D) or BRCA1 overexpression (Supplementary Figure S5E). Together, these data suggest that BRCA1 can positively regulate miR-593-5p expression in Cal-27 cells.

**Figure 5:**
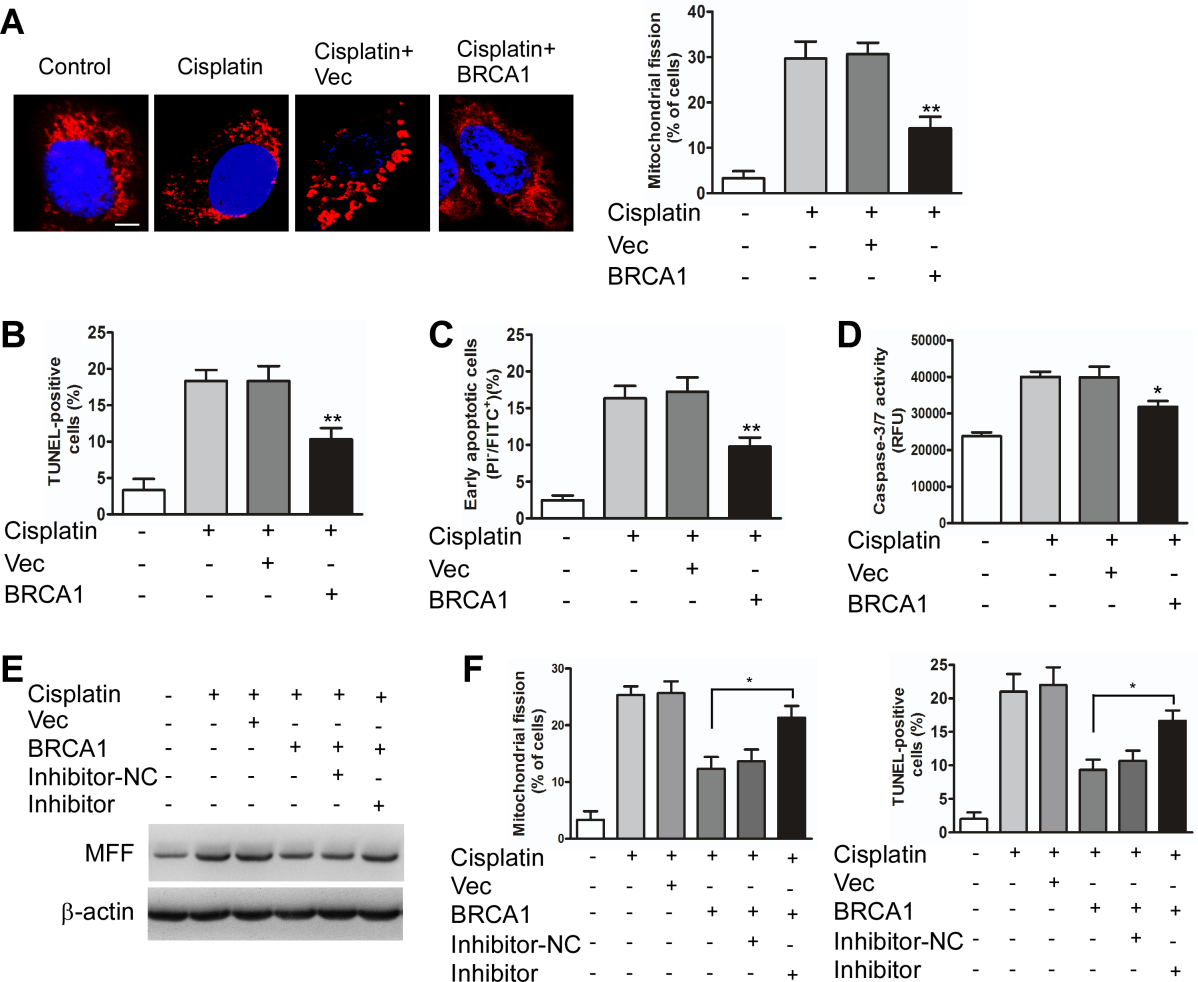
BRCA1 inhibits mitochondrial fission and apoptosis through miR-593-5p and MFF in Cal-27 cells **A**, BRCA1 attenuated mitochondrial fission in Cal-27 cells under cisplatin treatment. Cal-27 cells transiently transfected with BRCA1 expressing plasmids or vector control (Vec) were treated with cisplatin for 24h. Scale bar equals 3 μm. **P < 0.001 versus cisplatin alone. **B**, **C** and **D**, Apoptosis was detected using TUNEL, flow cytometry, and caspase-3/7 activity assays. *P < 0.01 versus cisplatin alone; **P < 0.001 versus cisplatin alone. **E**, The knockdown of miR-593-5p leads to the attenuation of the BRCA1 inhibitory effect on MFF protein levels under cisplatin treatment. Cal-27 cells stably expressing BRCA1 or vector control (Vec) were transfected with miR-593-5p inhibitors or inhibitor-negative control (inhibitor-NC). MFF levels were analyzed using immunoblotting. **F**, Mitochondrial fission and apoptosis were detected via staining with MitoTracker Red and TUNEL.*P < 0.01.

The role of BRCA1 in miR-593-5p expression led us to question whether the BRCA1–miR-593-5p–MFF axis is functionally related to mitochondrial fission and cisplatin sensitivity in TSCC cells. Cisplatin-induced mitochondrial fission and apoptosis were attenuated by BRCA1 (Figure 5A-D) in a manner that was dependent on the level of MFF protein (Supplementary Figure S5F) in Cal-27 cells. The knockdown of miR-593-5p attenuated the BRCA1 inhibitory effect on the level of MFF protein, mitochondrial fission and apoptosis induced by cisplatin(Figure 5E and 5F, Supplementary Figure S5G). We also found that BRCA1 was downregulated after cisplatin treatment and that exogenous BRCA1 attenuated mitochondrial fission and apoptosis as well as the miR-593-5p level and MFF protein level in Scc-9 cells (Supplementary Figure S6A-D). Taken together, BRCA1, miR-593-5p and MFF constitute an axis that regulates mitochondrial fission and cisplatin sensitivity in TSCC cells.

Meanwhile, we tested whether BRCA1–miR-593-5p–MFF axis-dependent regulation was sensitivity to other DNA-damaging agents. We found that MFF protein expression was upregulated but the expression of miR-593-5p and BRCA1 protein was downregulated when Cal-27 and Scc-9 cells were treated with adriamycin (ADR) or camptothecin(CPT) (Supplementary Figure S7A). In addition, we found that overexpression of BRCA1 could attenuate the mitochondrial fission and apoptosis induced by ADR or CPT(Supplementary Figure S7B and S7C). These data suggest that chemosensitivity to other DNA-damaging agents could also be regulated by the BRCA1–miR-593-5p–MFF axis in TSCC cells.

### Tongue squamous cell carcinoma xenografts

We established three groups of TSCC xenografts to investigate whether the BRCA1–miR-593-5p–MFF axis could influence the apoptosis and cisplatin sensitivity of TSCC cells *in vivo*.

**Figure 6:**
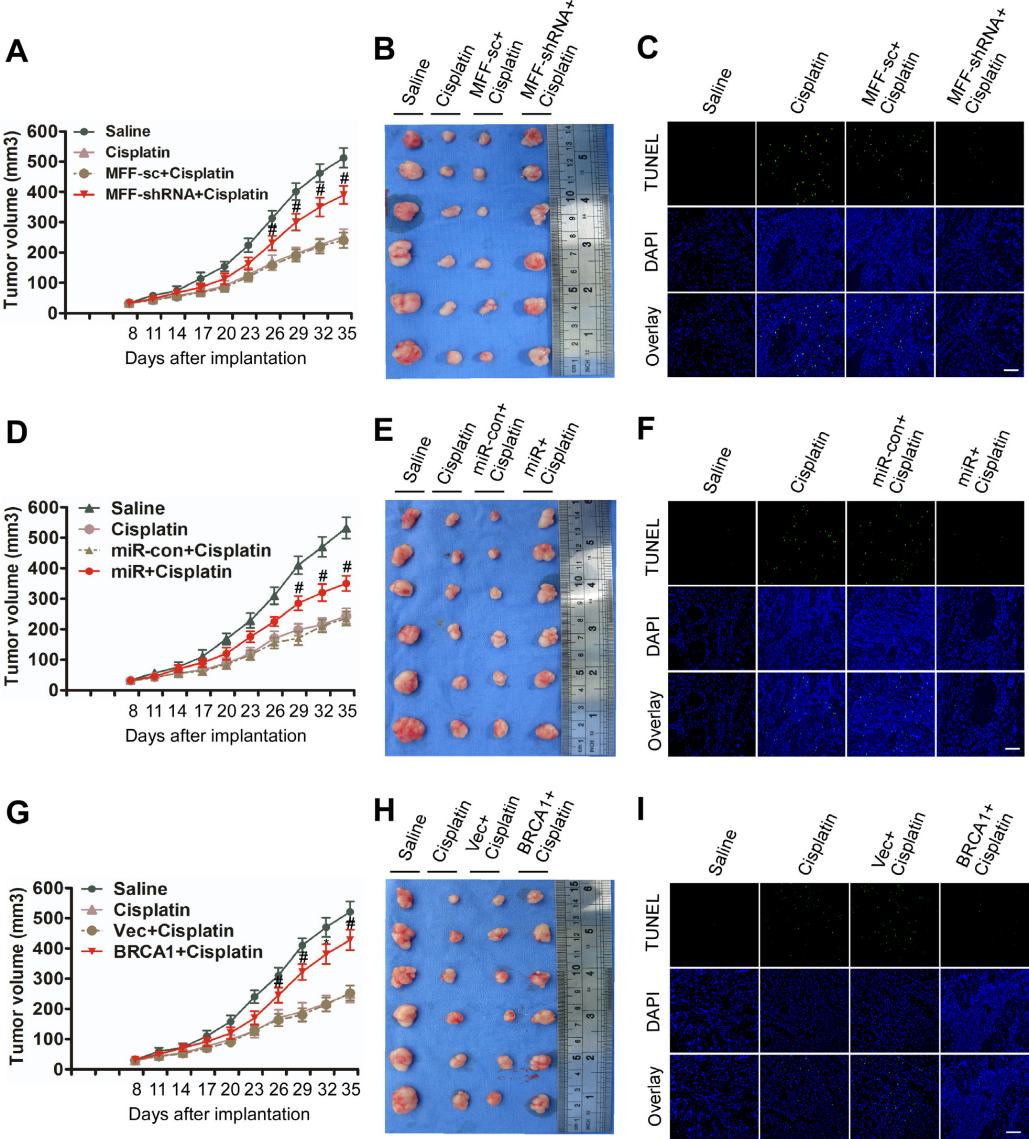
BRCA1–miR-593-5p–MFF axis attenuates the cisplatin-induced inhibition of tumor growth in Cal-27 cell xenografts in BALB/c-nu mice A, B, C, BALB/c-nu mice bearing Cal-27 cells with the stable expression of MFF shRNA or its scramble form (sc) were treated with saline or cisplatin. (**A**) Tumor growth curves for Cal-27 tumors treated with saline or cisplatin. (**B**) Representative photomicrographs of tumors from each group at day 35. (**C**) Apoptosis was detected via TUNEL assay. n=6 for each group. For TUNEL assay, n=24 slices from 6 xenograft tumors were sampled per group. Bar=20 μm; ^#^P < 0.05 versus cisplatin alone. D, E, F, BALB/c-nu mice bearing Cal-27 cells with the stable expression of miR-593-5p or its control (con) were treated with saline or cisplatin. (**D**) Tumor growth curves for Cal-27 tumors treated with saline or cisplatin. (**E**) Representative photomicrographs of tumors from each group at day 35. (**F**) Apoptosis was detected via TUNEL assay. n=6 for each group. For TUNEL assay, n=24 slices from 6 xenograft tumors were sampled per group. Bar=20 μm; ^#^P < 0.05 versus cisplatin alone. G, H, I, BALB/c-nu mice bearing Cal-27 cells with the stable expression of BRCA1 or empty vector (Vec) were treated with saline or cisplatin. (**G**) Tumor growth curves for Cal-27 tumors treated with saline or cisplatin. (**H**) Representative photomicrographs of tumors from each group at day 35. (**I**) Apoptosis was detected via TUNEL assay. n=6 for each group. For TUNEL assay, n=24 slices from 6 xenograft tumors were sampled per group. Bar=20 μm; ^#^P < 0.05 versus cisplatin alone; *P< 0.01 versus cisplatin alone.

First, we found that MFF knockdown attenuated the inhibition of the tumor burden induced by cisplatin (Figure 6A and 6B, Supplementary Figure S8A). Furthermore, MFF expression and apoptosis were attenuated by the stable expression of MFF shRNA in Cal-27 cells under cisplatin treatment (Figure 6C, Supplementary Figure S8A-C). These data suggest that MFF mediates the signal for apoptosis and cisplatin sensitivity *in vivo*. Cal-27 cells with stable miR-593-5p expression showed enhanced tumor growth in the presence of cisplatin (Figure 6D and 6E, Supplementary Figure S8D). We also found that miR-593-5p was upregulated but MFF and apoptosis were attenuated given the stable expression of miR-593-5p in xenografts after cisplatin treatment (Figure 6F, Supplementary Figure S8D-F). These results suggest that miR-593-5p inhibits apoptosis and cisplatin sensitivity *in vivo* by directly targeting MFF. Finally, in Cal-27 cells with stable BRCA1 expression, we observed that the inhibition of tumor growth (Figure 6G and 6H, Supplementary Figure S8G) was attenuated by BRCA1 in response to cisplatin treatment while BRCA1 expression and miR-593-5p levels were increased(Supplementary Figure S8H and 8I). MFF levels and apoptosis were attenuated by stable BRCA1 expression under cisplatin treatment (Figure 6I, Supplementary Figure S8G-I). These data suggest that BRCA1 could regulate apoptosis and cisplatin sensitivity *in vivo* through its downstream targets miR-593-5p and MFF. Taken together, the BRCA1-miR-593-5p–MFF axis regulates cisplatin sensitivity through the mitochondrial fission pathway *in vivo*.

### High MFF expression and low miR-593-5p or BRCA1 expression are associated with cisplatin sensitivity and good patient prognosis

**Figure 7:**
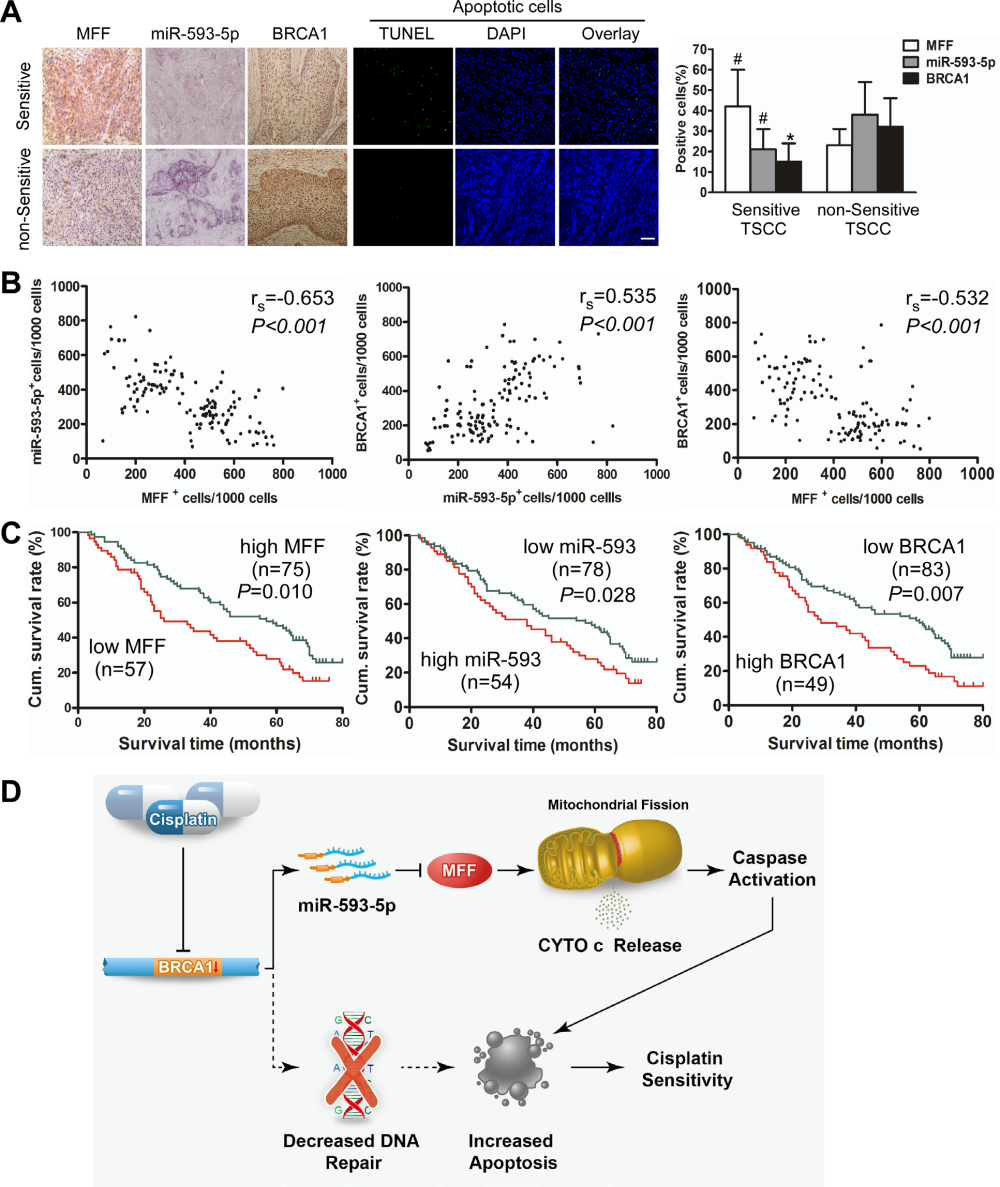
Expression of MFF, miR-593-5p and BRCA1 correlates with cisplatin sensitivity and survival of TSCC patients(TSCCs) **A**, MFF, miR-593-5p and BRCA1 expression and apoptosis were demonstrated in cisplatin-sensitive versus non-sensitive TSCCs. Left panel: MFF and BRCA1 expression were analyzed via immunohistochemistry; miR-593-5p expression was analyzed using in situ hybridization (×200). Apoptosis was detected using a TUNEL assay. Bar=20 μm. Right panel: Quantification of MFF, miR-593-5p and BRCA1 expression in cisplatin-sensitive versus non-sensitive TSCCs. ^#^P < 0.05; *P < 0.01. **B**, Associations between MFF, miR-593-5p and BRCA1 expression in TSCCs were analyzed via Spearman order correlation. **C**, Kaplan-Meier survival curves for TSCCs are plotted for MFF, miR-593-5p and BRCA1 expression, and survival differences were analyzed using a log-rank test. **D**, Model of the BRCA1–miR-593-5p–MFF axis in regulating mitochondrial fission and cisplatin sensitivity. The dotted line indicates the commonly accepted mechanism of BRCA1 regulation of cisplatin sensitivity, whereas the solid line represents the novel mechanism of BRCA1-mediated cisplatin sensitivity identified in the present study.

We evaluated the clinical significance of the BRCA1–miR-593-5p–MFF axis in cisplatin sensitivity as well as patient prognosis for TSCC. We performed a retrospective analysis of TSCC samples from 132 patients from three independent centers. According to previous studies [37], patients with a partial or complete response were defined as having cisplatin-sensitive tumors, whereas TSCC patients(TSCCs) with progressive disease or stable disease were defined as having non-sensitive or resistant tumors. Immunohistochemical staining and in situ hybridization demonstrated that MFF expression was higher and miR-593-5p and BRCA1 expression was lower in cisplatin-sensitive TSCC cells compared with non-sensitive cells (Figure 7A). Consequently, cisplatin-sensitive TSCC cells presented a higher percentage of apoptotic cells than non-sensitive cells (Figure 7A). There was a significant difference in the expression profiles of chemosensitive and non-sensitive TSCCs, as determined by the percentage of positive cells (Figure 7A). Additionally, a Spearman order correlation analysis showed that MFF expression in TSCC was inversely correlated with miR-593-5p (r_s_=−0.653, P<0.001) and BRCA1 levels (r_s_=−0.532, P < 0.001); however, miR-593-5p expression correlated with BRCA1 levels (r_s_=0.535, P<0.001; Figure 7B).

Next, we analyzed the association of MFF, miR-593-5p and BRCA1 expression with the clinicopathological status of TSCC patients (Table 1). No significant correlation was observed between MFF, miR-593-5p or BRCA1 expression and sex, age, lymph node status or clinical stage. However, MFF, miR-593-5p and BRCA1 expression were significantly associated with cisplatin sensitivity. Tumors with cisplatin sensitivity expressed higher levels of MFF and lower levels of miR-593-5p and BRCA1. Moreover, we evaluated the correlation between MFF, miR-593-5p and BRCA1 expression and patient overall survival (OS). A univariate Cox regression analysis indicated that the patients with TSCC and a high MFF expression level or low miR-593-5p or BRCA1 levels had a longer OS (Table 2 and Figure 7C). The cumulative survival rate at 60 months was 46.67%, 44.87% and 45.78% in patients with high MFF, low miR-593-5p and low BRCA1 expression, respectively; this rate was only 24.56%, 25.93% and 22.45% in those with low MFF, high miR-593-5p and high BRCA1 expression, respectively (Table 1). Furthermore, a multivariate Cox regression analysis revealed that the high-level expression of MFF and low-level expression of BRCA1 is an independent prognostic factor for good OS in patients with TSCC (Table 2). Together, these data suggest that the BRCA1–miR-593-5p–MFF axis correlates with cisplatin sensitivity and patient OS in TSCC.

**Table 1:** Correlation among clinicopathological status and the expression of MFF, miR-593-5p or BRCA1 in TSCC patients

Characteristics	MFF(%)		P	miR-593-5p(%)		P	BRCA1(%)		P
	No. of low Expression	No. of high Expression		No. of low Expression	No. of high Expression		No. of low Expression	No. of high Expression	
Sex			0.636			0.987			0.093
Male	32(45.1)	39(54.9)		42(59.2)	29(40.8)		40(56.3)	31(43.7)	
Female	25(41.0)	36(59.0)		36(59.0)	25(41.0)		43(70.5)	18(29.5)	
Age			0.321			0.650			0.337
<50	23(48.9)	24(51.1)		29(61.7)	18(38.3)		27(57.4)	20(42.6)	
≥50	34(40.0)	51(60.0)		49(57.6)	36(42.4)		56(65.9)	29(34.1)	
Node metastasis			0.866			0.067			0.076
NO	25(42.4)	34(57.6)		40(67.8)	19(32.2)		42(71.2)	17(28.8)	
N+	32(43.8)	41(56.2)		38(52.1)	35(47.9)		41(56.2)	32(43.8)	
Clinical stage			0.712			0.077			0.693
III	36(44.4)	45(55.6)		43(53.1)	38(46.9)		52(64.2)	29(35.8)	
IV	21(41.2)	30(58.8)		35(68.6)	16(31.4)		31(60.8)	20(39.2)	
Cisplatin			0.001			0.046			0.001
Sensitive	19(27.1)	51(72.9)		47(67.1)	23(32.9)		59(84.3)	11(15.7)	
Non-sensitive	38(61.3)	24(38.7)		31(50.0)	31(50.0)		24(38.7)	38(61.3)	
Status(60 months)			0.009			0.027			0.007
Survival	14(28.6)	35(71.4)		35(71.4)	14(28.6)		38(77.6)	11(22.4)	
Death	43(51.8)	40(48.2)		43(51.8)	40(48.2)		45(54.2)	38(45.8)	

**Table 2:** Univariate and multivariate analysis of factors associated with overall survival of patients with TSCC

Vavirable	Cases number	HR(95%C1)	P
Univariate analysis Sex Male vs Female	71/61	1.067(0.572-1.990)	0.813
Age(years) <50 vs ≥250	47/85	1.224(0.798-1.877)	0.312
Node metastasis N0 vs N +	59/73	1.461(1.014-2.105)	0.037
Clinical stage III VS IV	81/51	2.042(1.416-2.945)	0.000
Cisplatin Sensitive vs Non-sensitive	70/62	0.710(0.516-0.976)	0.043
MFF Low vs High	57/75	1.621(1.194-2.201)	0.010
miR-593-5p Low vs High	78/54	1.545(1.097-2.176)	0.028
BRCAI Low vs High	83/49	1.704(1.225-2.370)	0.007
Multivariate analysis Node metastasis NO vs N+	59/73	1.472(1.031-2.101)	0.041
Clinical stage III VS IV	81/51	2.325(1.476-3.662)	0.000
Cisplatin Sensitive vs Non-sensitive	70/62	0.527(0.358-0.776)	0.035
MFF Low vs High	57/75	1.837(1.105-3.175)	0.017
BRCAI Low vs High	83/49	2.053(1.284-3.283)	0.007

## DISCUSSION

The present study shows that MFF regulates mitochondrial fission and consequent cisplatin sensitivity. miR-593-5p, which is downregulated in response to cisplatin exposure, can inhibit MFF expression as well as mitochondrial fission by targeting MFF. We explored the mechanism by which miR-593-5p expression is regulated and found BRCA1 can transactivate miR-593-5p expression and inhibit MFF expression and the consequent mitochondrial fission through transcriptionally targeting miR-593-5p. Our results reveal a novel regulatory model of mitochondrial fission that affects cisplatin sensitivity via miRNA and BRCA1 in cancer cells.

In vertebrates, the target specificity of miRNAs is regulated by the requirement of small sequence complementarity between bases 2 and 8 of the miRNA and a corresponding seven-nucleotide sequence in the 3′UTR of the target mRNA [38]. The ability of a single miRNA to affect the expression of a wide variety of proteins has led to increased interest in miRNAs as mediators of the cellular response to cisplatin stimulation, and numerous miRNAs have been identified [33, 39]. However, no miRNAs have been identified as regulating mitochondrial fission and consequent cisplatin sensitivity. We found that MFF is a direct target of miR-593-5p and that miR-593-5p inhibits cisplatin sensitivity in TSCC through its suppression of MFF expression.

It has been demonstrated that approximately 50% of miRNAs are predicted to be expressed from the introns of protein-coding transcripts [40]. Accordingly, intronic miRNAs may be transcribed as part of their host genes or transcribed independently using their own promoters. Ongoing studies have revealed that one-third of intronic miRNAs have transcription initiation regions that are separate from the promoters of their host genes [41]. Strikingly, miR-593-5p is an intronic miRNA in the SND1 gene; we identified the binding site of BRCA1 to be within the SND1 gene, and BRCA1 consistently influenced the expression of miR-593-5p, but not of SND1, in our findings.

The chemotherapeutic drug cisplatin primarily acts against cancer by damaging DNA and is used in the treatment of many solid tumors. BRCA1 is a tumor suppressor gene located on chromosome 17q21 that has been intensively investigated as participating in the repair of cisplatin-induced DNA double-strand breaks [42]. Accordingly, a decrease in BRCA1 expression leads to a decreased proficiency in DNA repair, increased cisplatin sensitivity and improved survival in non-small cell lung cancer [43, 44], breast cancer [45], advanced esophageal squamous cell carcinoma [46] and ovarian cancer [47] patients. However, it is currently unknown whether BRCA1 expression correlates with cisplatin sensitivity in TSCC. In this study, we found that cisplatin decreased the level of BRCA1 in TSCC patients and that reduction of BRCA1 was positively correlated with overall survival. Notably, the mechanism of BRCA1 downregulation by cisplatin remains poorly understood, a few previous studies demonstrated that BRCA1 can be downregulated at translation [48, 49] or transcription levels [50, 51] by cisplatin. Recent studies found BRCA1 was a direct targets of miR-638 [48] and miR-9 [49] and BRCA1 protein decreased upon cisplatin treatment. However, the mechanism of BRCA1 mRNA downregulation has not been clear, there may be a TP53-sensitive component [50] or EZH2-dependent [51] which need to be verified in the further studies.

In addition to its role in DNA repair, BRCA1 also has multiple roles in cellular functions, such as transcriptional regulation. The transcriptional activity of the C-terminal region of BRCA1 was first identified when it was fused with the GAL4 DNA-binding domain [52]. Recent studies have firmly established the role of BRCA1 as a transcriptional activator and transcriptional repressor [53]. A recent study identified the direct transcriptional targets of BRCA1 by combining gene expression data with BRCA1 binding sites detected using ChIP-chip analysis. However, the results suggested that only 44 out of the 1,294 transcriptional targets may be directly regulated by BRCA1 [54]. Whether BRCA1 binds to DNA directly or indirectly has been extensively debated, but it is accepted that BRCA1 interacts with DNA not only to repair damaged DNA but also to regulate transcription. Our results suggest that BRCA1 has a novel role in the regulation of mitochondrial fission and cisplatin sensitivity through transactivating miR-593-5p expression in TSCC cells.

The present study reveals a link between the BRCA1-miR-593-5p-MFF axis and the mitochondrial fission program of TSCC (Figure 7D). Future studies are needed to elucidate how this pathway is integrated into the DNA repair pathway and its relationship with other intrinsic apoptotic factors. Notably, clinical evidence suggests that BRCA1, miR-593-5p and MFF levels predict cisplatin sensitivity, and the modulation of this axis may provide a therapeutic approach for upregulating cisplatin sensitivity.

## MATERIALS AND METHODS

### Cell culture

The human tongue cancer cell lines Cal-27 and Scc-9 were purchased from the American Type Culture Collection. Cal-27 cells were cultivated in Dulbecco's modified Eagle's medium (Gibco, Rockville, MD, USA) supplemented with 10% fetal bovine serum (Invitrogen, Carlsbad, CA, USA). Scc-9 cells were cultivated in Dulbecco's modified Eagle's medium-F12 (Gibco) supplemented with 10% fetal bovine serum.

### ChIP assays

ChIP assays were performed as previously described with some modifications [15, 54]. The antibodies used for immunoprecipitation were rabbit IgG (sc-805, Santa Cruz) and BRCA1 (ab16780; Abcam). In brief, Cal-27 cells (5×10^6^) were washed with PBS and incubated for 10 min with 1% formaldehyde at room temperature. The cross-linking was halted with 0.1 M glycine for 5 min. The cells were washed twice with PBS and lysed for 1 h at 4°C in a lysis buffer and then sonicated into chromatin fragments with an average length of 500-800 bp as assessed via agarose gel electrophoresis. The samples were precleared with Protein-A agarose (Roche) for 1 h at 4°C on a rocking platform, and 5 μg of specific antibodies were added and rocked overnight at 4°C. Immunoprecipitated DNA was purified using the QIAquick PCR purification kit (Qiagen) according to the manufacturer's protocol. The final ChIP DNA was then used as a template in qPCR reactions using primers that encompass ten possible BRCA1 binding sites of the homo miR-593-5p promoter. The primers are presented in Supplementary Table S1. The specificity of the PCR amplification was confirmed via agarose gel electrophoresis.

### Transfections

MFF siRNAs (E-018261) and BRCA1 siRNAs (E-003461) were obtained from GE Dharmacon. miR-593-5p mimics and inhibitors were obtained from Ribobio (Guangzhou, China). Cells were transfected using Lipofectamine 2000 (Invitrogen).

### Plasmid construction and establishment of stable cell lines

An MFF shRNA retrovirus vector (pSR-puro-MFF1 shRNA, 37247) [55] and a BRCA1 (pBABE-puro HA BRCA1, 14999) [56] retrovirus vector were obtained from Addgene (MA, USA). BRCA1 was also cloned into pcDNA3.1 to generate pcDNA-BRCA1. The primers to amplify BRCA1 were as follows: forward: 5′-TAGATATCATGGATTTATCTGCTCTTCGC-3′ and reverse: 5′-CTCTCGAGTCAGTAGTGGCTGTGGGGGA-3′. MFF CDS region was amplified by PCR using the forward primer 5′-CAGGATCCATGAGTAAAGGAACAAGCAGTGA-3′, the reverse primer: 5′-TACTCGAGCTAGCGGCGAAACCAGAGC-3′, and then cloned into pcDNA3.1.

Recombinant retrovirus was generated by co-transfecting pSuper-Retro-Puro carrying the shRNA expression cassette with helper plasmid pIK in 293T cells for 48 h. The viral supernatants were collected, filtered, mixed with fresh complete medium (1:1) and 4 μg/mL of polybrene (Sigma, St Louis, Missouri, USA), and then added to Cal-27 or Scc-9 TSCC cells. The stably infected cells were selected with 2μg/mL of Puromycin (Sigma, St Louis, Missouri, USA) for two weeks.

Lentiviral plasmid (pLVX-mCMV-tdTomato-PGK-Puro) delivering miR-593 precursors were bought from BioWit Technologies (Shenzhen, China). The viruses were amplified in HEK293 cells. Lentiviral infection of the Cal-27 cell line was performed as previously described [57].

### Luciferase reporter assay

We cloned MFF expression cassette containing miR-593-5p targeting site (wide type or mutated) into the pGL3-Control plasmid downstream of the luciferase reporter gene. In addition, we cloned miR-593-5p promoter region containing the BRCA1 binding site sequence (wild type or mutated) into the pGL3-Basic plasmid upstream of the luciferase reporter gene. Luciferase activities were measured using a luciferase assay kit (Promega, Madison, WI, USA), and the target effect was expressed as the relative luciferase activity of the reporter vector with the target sequence over that without the target sequence.

### Target protector preparation and transfection

The target protector (TP) was designed and named as others described [15, 34]. In brief, the MFF-TP miR-593-5p sequence was 5′-CGACATAAGTGCAGACTCGTCAAGA-3′. The MFF-TP control was 5′-CGAGATAACTCCACACTCCTCAAGA-3′. The sequences were obtained from Gene Tools. Transfection of the target protector was performed using an Endo-Porter kit (Gene Tools) according to the manufacturer's instructions.

### Isolation of mitochondria and cytosol

Subcellular fractions were prepared as described previously [16]. In brief, the cells were washed twice with cold PBS, and the pellet was suspended in 0.2 ml of buffer (20 mM HEPES pH 7.5, 10 mM KCl, 1.5 mM MgCl2, 1 mM EGTA, 1 mM EDTA, 1 mM dithiothreitol (DTT), 0.1 mM phenylmethylsulfonyl fluoride (PMSF), and 250 mM sucrose) containing a protease inhibitor cocktail. Then, the cells were homogenized with 12 strokes in a Dounce homogenizer, followed by centrifugation twice at 750 g for 5 min at 4°C to collect nuclei and debris. The mitochondria-enriched heavy membrane pellet was collected with further centrifugation at 10,000 g for 15 min at 4°C; the supernatants were collected as the cytosolic fractions.

### Apoptosis assay

Cal-27 and Scc-9 cells were treated with the IC_50_ of cisplatin(Sigma, USA) for 24 hours for an apoptosis assay [37, 58]. Additionally, Cal-27 and Scc-9 cells were treated with 2×10^−6^ M adriamycin(ADR) (Sigma, USA) or 15×10^−6^ M camptothecin(CPT) (Sigma, USA) for 24 hours for an apoptosis assay. TUNEL technology was performed using a kit from Roche (Cat.No.11684795910). The detection procedures were in accordance with the kit instructions. Sections were examined with an ImagerZ1 microscope (Zeiss, Jena, Germany). An investigator blind to the treatment quantified 20 random fields of samples. Caspase-3/7 activity was determined using an Apo-ONE® Homogeneous Caspase-3/7 assay kit from Promega according to the manufacturer's protocol. Flow cytometry was performed using Annexin V and propidium iodide double staining (Sigma-Aldrich).

### MTT assay

To monitor the IC50 of cisplatin, Cal-27 and Scc-9 cells were treated with cisplatin atdifferent concentrations for 24 hours. An MTT assay was performed as described previously [37]. The data were analyzed with the software origin 7.5 (OriginLab, Northampton, MA, USA) to fit a sigmoidal curve. The IC_50_ is considered to be the cisplatin concentration that reduces cell proliferation by 50%. The IC50 values of cisplatin for Cal-27 and Scc-9 cells were 8×10^−6^ and 1.8×10^−5^ M, respectively.

### Immunofluorescence staining

Cells on coverslips were stained for MFF and cytochrome c(CYTO c). After mitochondrial staining, the cells were incubated with primary antibodies against MFF (ab81127, Abcam) or cytochrome c (sc-13560, Santa Cruz) and then incubated with rhodamine- or FITC-conjugated secondary antibodies (Invitrogen). The coverslips were counterstained with 46-diamidino-2-phenyl indole and imaged under a confocal microscope TCS SP5 (Lecia, Solms, Germany).

### Mitochondrial staining and analysis of mitochondrial fission

Mitochondrial staining was performed as described previously [15, 16], with modifications. Briefly, cells were plated onto coverslips and treated under different conditions. Then, they were stained for 30 min with 0.1 μM MitoTracker Red CMXRos (Molecular Probes). The mitochondria were imaged using a laser-scanning confocal TCS SP5 microscope (Lecia, Solms, Germany). The assessment and quantification of mitochondrial morphology were performed as described previously [59]. Briefly, the extent of mitochondrial fission was analyzed on a cell-to-cell basis. Mitochondria fission was calculated as the percentage of cells with fragmented mitochondria relative to the total number of cells, which were randomly selected and scored. A punctiform mitochondrial phenotype was scored as a fragmented mitochondrion when at least 90% of its tubular mitochondria were disintegrated. At least 200 randomly selected cells in multiple fields were assessed.

### Quantitative real-time PCR (qRT-PCR)

Total RNA was prepared using TRIzol reagent (Invitrogen). The quantitative detection of MFF, SND1 and β-actin was performed via qRT-PCR using SYBR Green Real-time PCR Master Mix (ReverTra Ace, Toyobo) and a LightCycler 480 (Roche, Basel, Switzerland) according to the manufacturer's instructions. The sequences of MFF primers were 5′-CACCACCTCGTGTACTTACGC-3′ (forward) and 5′-CCGCTCTCTTTTTAGTCTGCC-3′ (reverse). The sequences of the SND1 primers were 5′-CAAATCAGGAAGAAACATCAAAGAC-3′ (forward) and 5′-AATCACATAATCAACAGTTGGACAG-3′ (reverse). The sequences of the β-actin primers were 5′-AGCCTCGCCTTTGCCGATCC-3′ (forward) and 5′-ACATGCCGGAGCCGTTGTCG-3′ (reverse). The primers for miR-593-5p and U6 detection assays were purchased from Ribobio.

### Western blotting

Immunoblotting was performed as previously described [37]. Briefly, cells were lysed for 0.5 h at 4°C in a RIPA Buffer (R0278, Sigma) containing a protease inhibitor cocktail. Protein extracts were resolved through 8% SDS-polyacrylamide gel electrophoresis, transferred to polyvinylidene difluoride membranes (BioRad, Berkeley, CA, USA), probed with antibody against human MFF (ab81127), DRP1 (ab56788), FIS1 (ab71498), TOM20(ab78547), BRCA1 (ab16780; Abcam), MFN1 (sc-50330), MFN2 (sc-50331), OPA1 (sc-393296), COXIV(sc-376731), cytochrome c (sc-13560) (Santa Cruz), β-actin (Proteintech, Chicago, IL, USA) or Tubulin (sc-53646) and then with a peroxidase-conjugated secondary antibody (Proteintech); they were visualized via chemiluminescence (GE, Fairfield, CT, USA).

### *In situ* hybridization

In situ hybridization was performed as previously described [37] according to the manufacturer's protocol (Exiqon, Vedbaek, Denmark). Briefly, after demasking, miR-593-5p was hybridized to 5′ DIG-labeled LNAprobes. Then, the digoxigenins were recognized via a specific anti-DIG antibody that is directly conjugated to alkaline phosphatase. The nuclei were counterstained with Kernechtrot Solution (N3020, Sigma). In all, 5×200 tumor cells were counted randomly in each section. The sections with more than 300 miR-593-5p-positive cells were considered to have high miR-593-5p expression.

### Immunohistochemistry

For immunohistochemistry [37], TSCC sections were incubated with MFF (ab81127) and BRCA1 (ab16780, Abcam) antibodies at 4°C overnight. The sections were then treated with a secondary antibody, followed by further incubation with streptavidin-horseradish peroxidase complex. Diaminobenzidine (Dako, Carpinteria, CA, USA) was used as a chromogen, and the nuclei were counterstained with hematoxylin. A total of 5×200 tumor cells were counted in each section. Sections with more than 350 MFF- or BRCA1-positive cells were considered to have high MFF or BRCA1 expression.

### Patient and tissue samples

Specimens of locally advanced TSCCs (n=132) were obtained from three independent centers, including the Department of Oral and Maxillofacial Surgery of Sun Yat-sen Memorial Hospital (n=53), the Affiliated Hospital of North Sichuan Medical College (n=45) and the West China Hospital (n=34) between Jan 1, 2002, and Dec 31, 2008. The patients were treated with cisplatin prior to surgery. According to the ‘Response Evaluation Criteria in Solid Tumors’ of the World Health Organization, TSCCs with progressive disease or stable disease response was designated to be cisplatin-resistant or non-sensitive TSCC, whereas TSCCs that showed a partial or complete response was determined to be cisplatin-sensitive TSCC. The tissues were obtained from the respective pathology departments, and histological diagnosis and scoring of all the cases were performed by two independent pathologists. Survival time was calculated from the date of surgery to the date of death or to the last follow-up. The date of death was obtained from patient records or through follow-up telephone calls. This study was approved by the institutional ethical review boards of three hospitals, and written informed consent was obtained from all patients.

### Tumor xenografts

A TSCC xenograft mouse model was used to evaluate *in vivo* cisplatin sensitivity. Male BALB/c-nu mice 4 to 6 weeks old were prepared for tumor implantation. All animals were maintained in a sterile environment on a daily 12 h light/12 h dark cycle. Cal-27 cells with stable expression of MFF, miR-593-5p and BRCA1 were used. After resuspension in 150 μL of PBS, Cal-27 cells (5×10^6^/mouse) were injected subcutaneously into the flanks of the nude mice. One week after implantation, when the tumor became palpable at a size of ~2 mm in diameter, cisplatin (5 mg/kg) was administered via intraperitoneal injections every three days from days 8 to 32. Tumor volume was calculated at the day of cisplatin injection according to the following formula: TV (mm^3^)=length × width^2^×0.5. At day 35, the primary tumors were carefully removed, imaged, and analyzed via immunohistochemistry, in situ hybridization, western blotting and qRT-PCR.

### Statistics

All statistical analyses were performed using SPSS 19.0. Student's t-test and the Chi-square test were used to analyze the relationship between MFF, miR-593-5p and BRCA1 expression and clinicopathological characteristics. To measure the association between pairs of variables, Spearman order correlations were performed. Kaplan-Meier survival curves were plotted, and a log-rank test was performed. All experiments for cell cultures were performed in at least three independent experiments. The data are expressed as the means ± SEM. P<0.05 was considered significant.

## SUPPLEMENTARY MATERIAL FIGURES AND TABLE


