# Supplementary material for: Mitochondrial fission determines cisplatin sensitivity in tongue squamous cell carcinoma through the BRCA1–miR-593-5p–MFF axis
Source: Oncotarget. 2015 Mar 26;6(17):14885–904. doi: 10.18632/oncotarget.3659 (PMC4558123; doi:10.18632/oncotarget.3659)
Supplement: Supplementary file 1 [file oncotarget-06-14885-s001.pdf]

Mitochondrial fission determines cisplatin sensitivity in tongue squamous cell carcinoma through the BRCA1–miR-593-5p–MFF axis

Supplementary Material

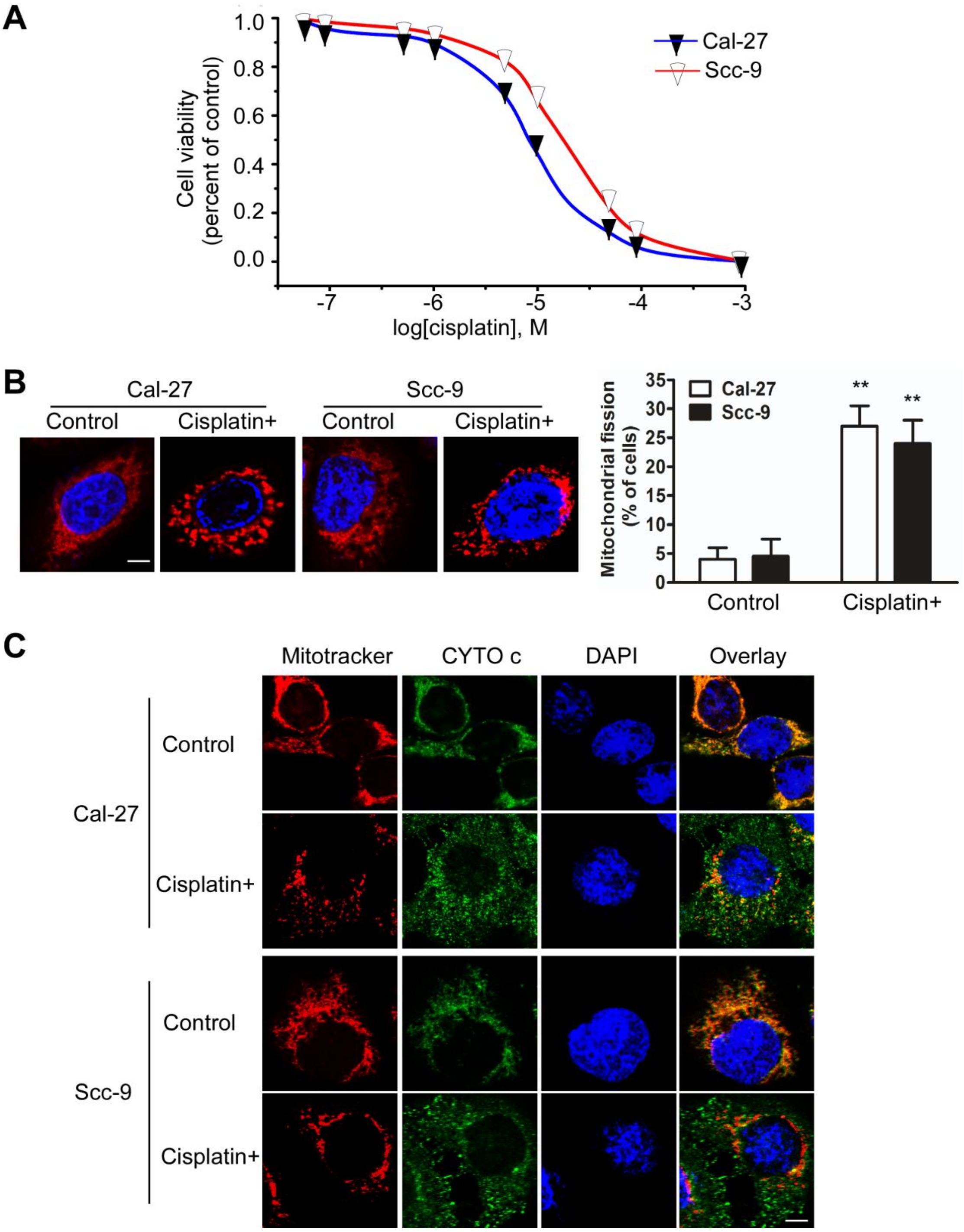

Supplementary Figure S1: Mitochondrial fission participates in TSCC cell

apoptosis after cisplatin treatment *in vitro*. A, The MTT assay was used to determine the cisplatin sensitivity of Cal-27 and Scc-9 cells. B, Mitochondrial fission is increased after cisplatin treatment. Cal-27 and Scc-9 cells were treated with the IC_{50} of cisplatin for 24 hours, respectively. Left panel: mitochondria were visualized via staining with MitoTracker Red. Scale bar equals 3 μm. Right panel: counting of cells with mitochondrial fission. $^{**}P < 0.001$versus no cisplatin treatment. C, Cytochrome c (CYTO c) distribution in mitochondria. Immunofluorescence analysis of CYTO c distribution in mitochondria without and with cisplatin treatment for 24 hours. The cells were labeled with MitoTracker (red), stained with an anti-CYTO c antibody, and monitored via FITC-labeled secondary antibody (green). The overlay of red and green yields an orange and/or yellow color. Scale bar equals 3 μm.

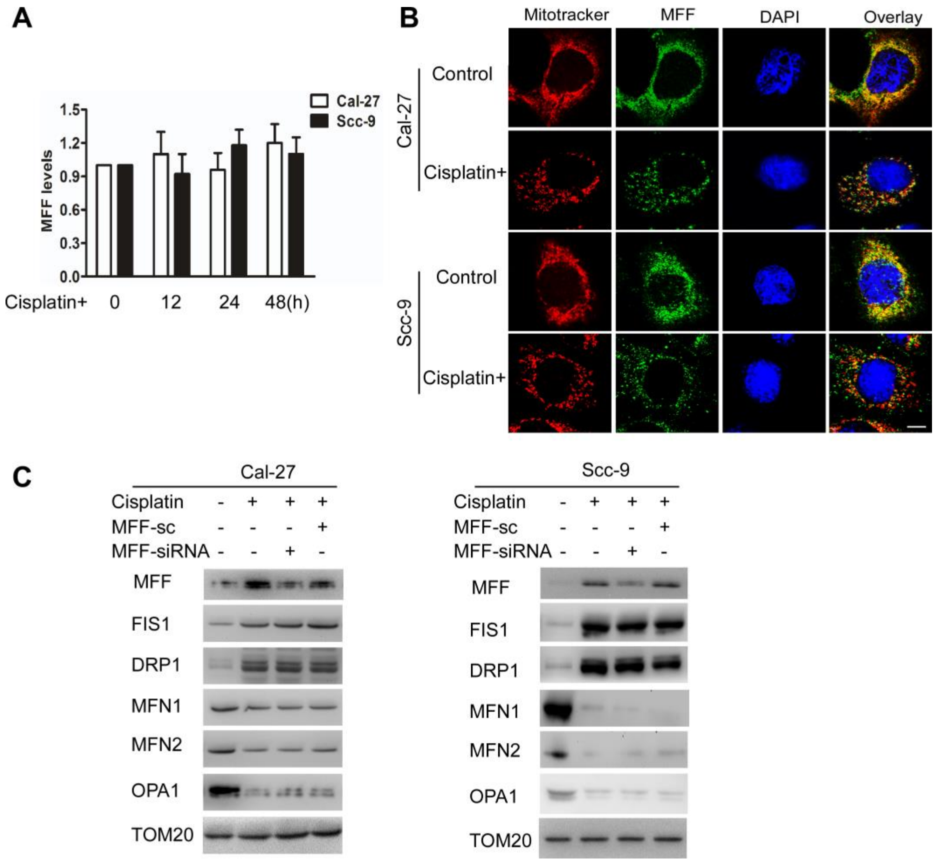

Supplementary Figure S2: MFF regulates mitochondrial fission and apoptosis in TSCC cells after cisplatin treatment. A, qRT-PCR was performed to analyze MFF mRNA levels under cisplatin treatment. The results are expressed as the means ± SEM of three independent experiments. B, Immunofluorescence analysis of MFF distribution in mitochondria with and without cisplatin treatment for 24 h. The cells were labeled with MitoTracker (red), stained with an anti-MFF antibody, and monitored using a FITC-labeled secondary antibody (green). The overlay of red and green yields an orange and/or yellow color. The scale bar equals 3 μm. C, Knockdown of MFF attenuated cisplatin-induced MFF protein levels. Cal-27 and Scc-9 cells were transfected with MFF small interfering RNA (siRNA) or its scramble form (sc) and then exposed to cisplatin for 24 hours. Detection of MFF, FIS1, DRP1, MFN1, MFN2 and OPA1 using immunoblotting.

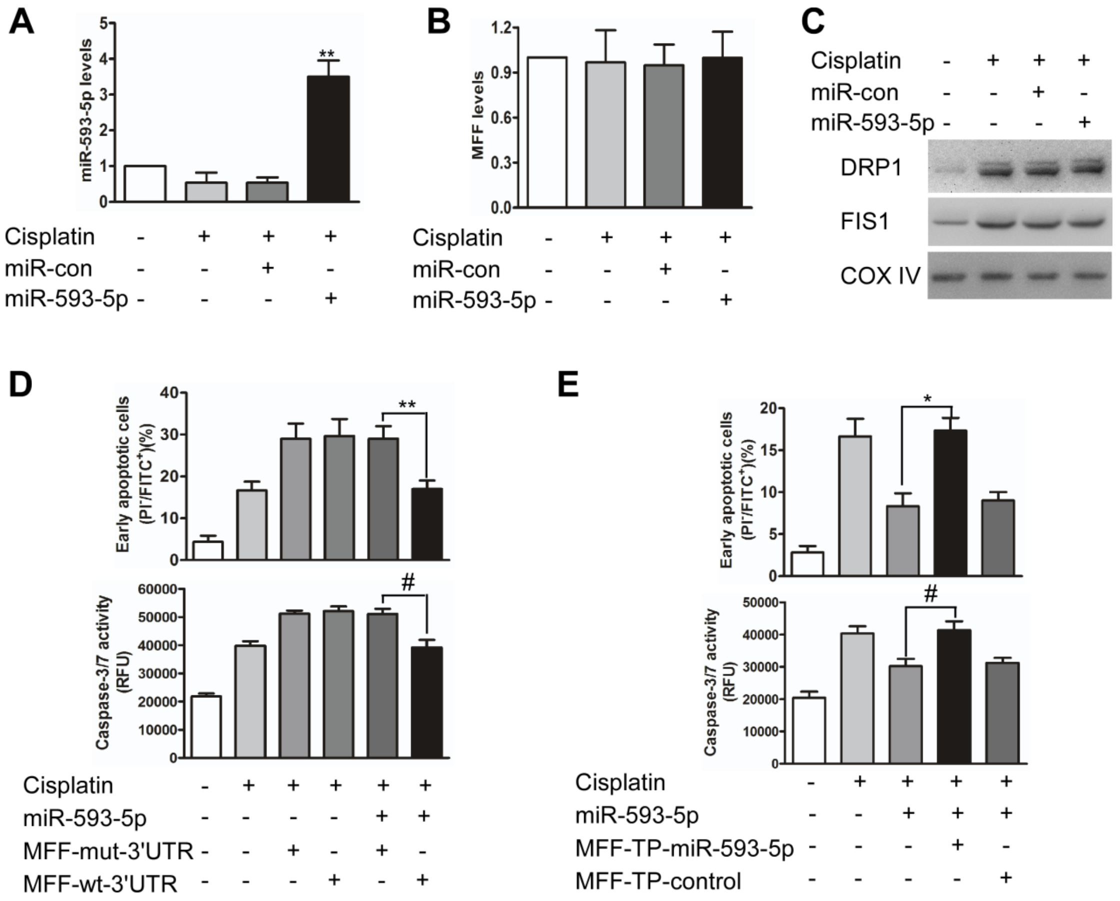

Supplementary Figure S3: miR-593-5p regulates apoptosis in Cal-27 cells by targeting the 3'UTR of MFF. A, miR-593-5p levels were detected using qRT-PCR. Cal-27 cells were transfected with miR-593-5p mimics or a miR-593-5p control (miR-con). After 24 h of transfection, the cells were treated with cisplatin for 24 h. The results are expressed as the means ± SEM of three independent experiments. ***P*< 0.001 versus cisplatin alone. B, MFF mRNA was detected using qRT-PCR in Cal-27 cells. The results are expressed as the means ± SEM of three independent experiments. C, DRP1 and FIS1 levels were analyzed via immunoblotting in Cal-27 cells. D, miR-593-5p attenuated apoptosis in the presence of MFF with wild type 3'UTR (MFF-wt-3'UTR) but not its mutated 3'UTR (MFF-mut-3'UTR). Cal-27 cells were transfected with miR-593-5p mimics along with pcDNA3.1 plasmid carrying a

wild-type (MFF-wt-3’UTR) or mutatated (MFF-mut-3’UTR) MFF expression cassette at miR-593-5p response element. Apoptosis were detected via flow cytometryand caspase-3/7 activity assays. $^{\#}P< 0.05$; $^{**}P< 0.001$. E, MFF target protector reduces the inhibitory effect of miR-593-5p on apoptosis. Cal-27 cells were transfected with miR-593-5p mimics, along with the target protector (MFF-TP-miR-593-5p) or the control (MFF-TP-control). Apoptosis were detected via flow cytometry and caspase-3/7 activity assays. $^{\#}P< 0.05$;$^{*}P< 0.01$.

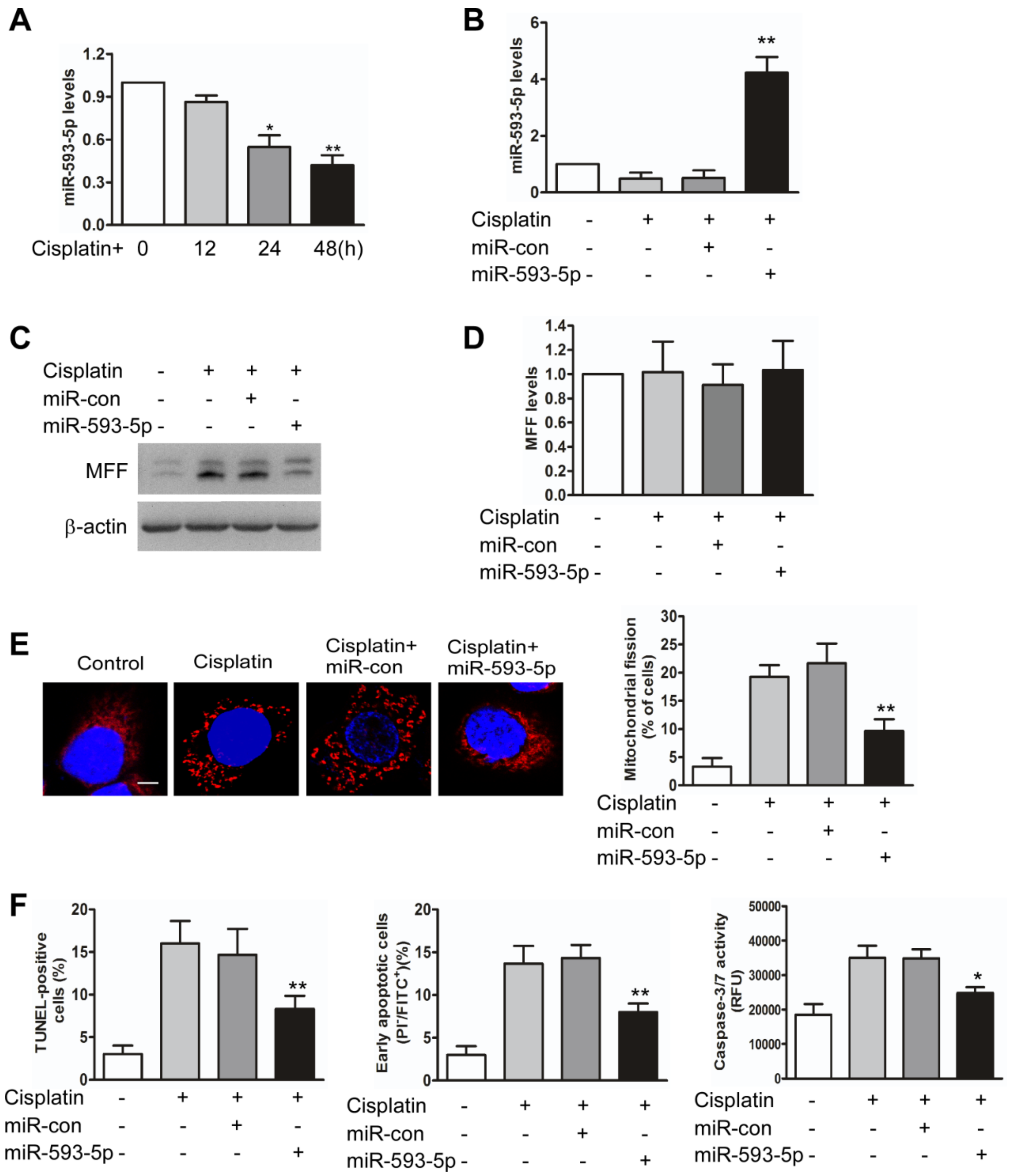

Supplementary Figure S4: miR-593-5p regulates mitochondrial fission and apoptosis in Scc-9 cells. A, qRT–PCR was performed to analyze miR-593-5p levels in Scc-9 cells under cisplatin treatment. The results are expressed as the means ± SEM of three independent experiments. **P*< 0.01 versus no cisplatin treatment; ***P*< 0.001 versus no cisplatin treatment. B, Forced expression of miR-593-5p attenuated the decrease in miR-593-5p levels after cisplatin treatment. Scc-9 cells were transfected with miR-593-5p mimics or miR-593-5p control (miR-con). After 24 h of transfection,

the cells were exposed to cisplatin and harvested 24 h after treatment for miR-593-5p analysis. The results are expressed as the means ± SEM of three independent experiments. $^{**}P < 0.001$ versus cisplatin alone. C, miR-593-5p attenuated MFF protein levels. MFF levels were analyzed using immunoblotting. D, qRT-PCR was performed to analyze MFF mRNA levels. The results are expressed as the means ± SEM of three independent experiments. E, miR-593-5p attenuated mitochondrial fission in Scc-9 under cisplatin treatment. Scale bar equals 3 µm. The results are expressed as the means ± SEM of three independent experiments. $^{**}P < 0.001$ versus cisplatin alone. F, Apoptosis was detected using TUNEL, flow cytometry, and caspase-3/7 activity assays. The results are expressed as the means ± SEM of three independent experiments. $^{*}P < 0.01$ versus cisplatin alone; $^{**}P < 0.001$ versus cisplatin alone.

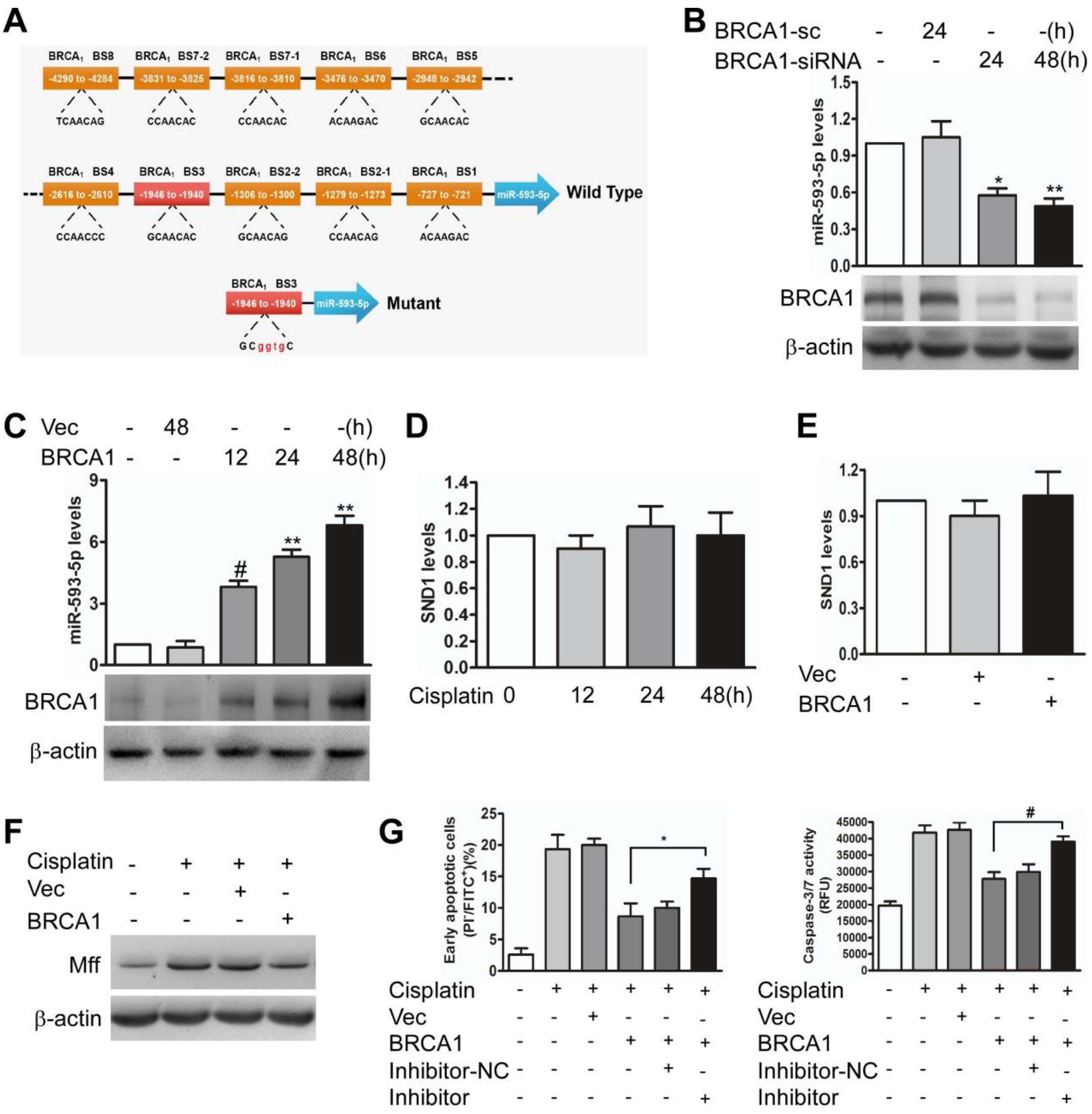

Supplementary Figure S5: BRCA1 transactivates miR-593-5p but not SND1. A, The homo miR-593-5p promoter region contains ten potential BRCA1 binding sites (jaspar.genereg.net). B, The knockdown of endogenous BRCA1 leads to a reduction in miR-593-5p levels. Cal-27 cells were transfected with BRCA1 siRNA or its scramble form (BRCA1-sc). miR-593-5p was detected using qRT-PCR (upper panel); BRCA1 was analyzed via immunoblotting (lower panel). The results are expressed as the means ± SEM of three independent experiments. *P< 0.01 versus scramble; **P< 0.001 versus scramble. C, The forced expression of BRCA1 stimulates miR-593-5p expression. Cal-27 cells were transfected with BRCA1 expressing plismids or empty

vector (Vec). miR-593-5p was detected using qRT–PCR (upper panel); BRCA1 was analyzed using immunoblotting (lower panel). The results are expressed as the means ± SEM of three independent experiments. $^{\#}P < 0.05$ versus empty vector; $^{**}P < 0.001$ versus empty vector. D, SND1 mRNA levels in Cal-27 cells exposed to cisplatin. Cal-27 cells were exposed to cisplatin and harvested at the indicated times for a qRT-PCR analysis of SND1. The results are expressed as the means ± SEM of three independent experiments. E, BRCA1 did not affect the SND1 mRNA levels. Cal27 cells were transfected with BRCA1 expressing plismids or empty vector(Vec) and harvested 24 h after infection for detecting SND1 mRNA via qRT-PCR. The results are expressed as the means ± SEM of three independent experiments. F, The forced expression of BRCA1 reduces MFF expression. Cal-27 cells were transiently transfected with BRCA1 expressing plismids or empty vector(Vec) for 24 h and then treated with cisplatin for 24 h. MFF expression was analyzed using immunoblotting. G, The knockdown of miR-593-5p leads to the attenuation of the BRCA1 inhibitory effect on apoptosis under cisplatin treatment. Cal-27 cells stably expressing BRCA1 or vector control (Vec) were transfected with miR-593-5p inhibitors or inhibitor-negative control (inhibitor-NC). Apoptosis were detected via flow cytometry and caspase-3/7 activity assays. $^{\#}P < 0.05$;$^{*}P < 0.01$.

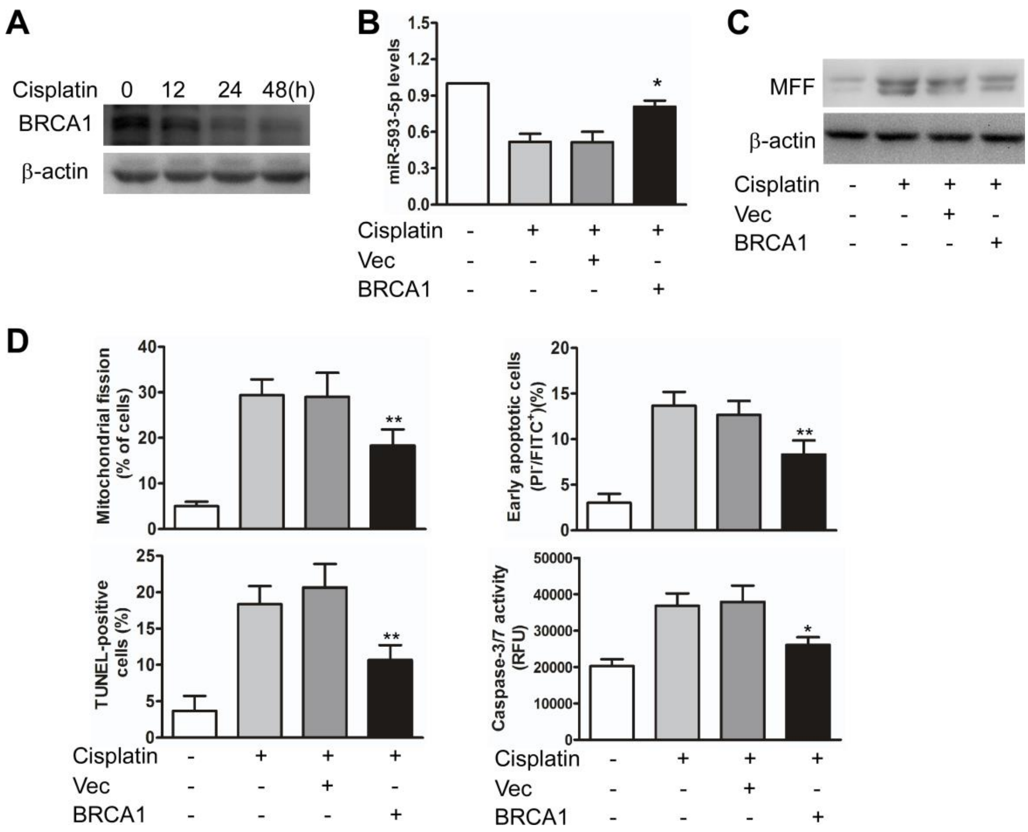

Supplementary Figure S6: BRCA1 regulates mitochondrial fission and apoptosis in Scc-9 cells. A, BRCA1 was analyzed using immunoblotting in Scc-9 cells under cisplatin treatment. B, BRCA1 attenuated the cisplatin-induced decrease of miR-593-5p. Scc-9 cells were transfected with BRCA1 expressing plismids or empty vector (Vec) for 24 h and then treated with cisplatin for 24 h. The results are expressed as the means ± SEM of three independent experiments. **P*< 0.01 versus cisplatin alone. C, BRCA1 attenuated the cisplatin-induced increase in MFF protein levels. MFF levels were analyzed via immunoblotting. D, Mitochondrial fission and apoptosis were detected via staining with MitoTracker Red, flow cytometry, TUNEL, and caspase-3/7 activity assays. The results are expressed as the means ± SEM of three independent experiments. **P*< 0.01 versus cisplatin alone; ***P*< 0.001 versus cisplatin alone.

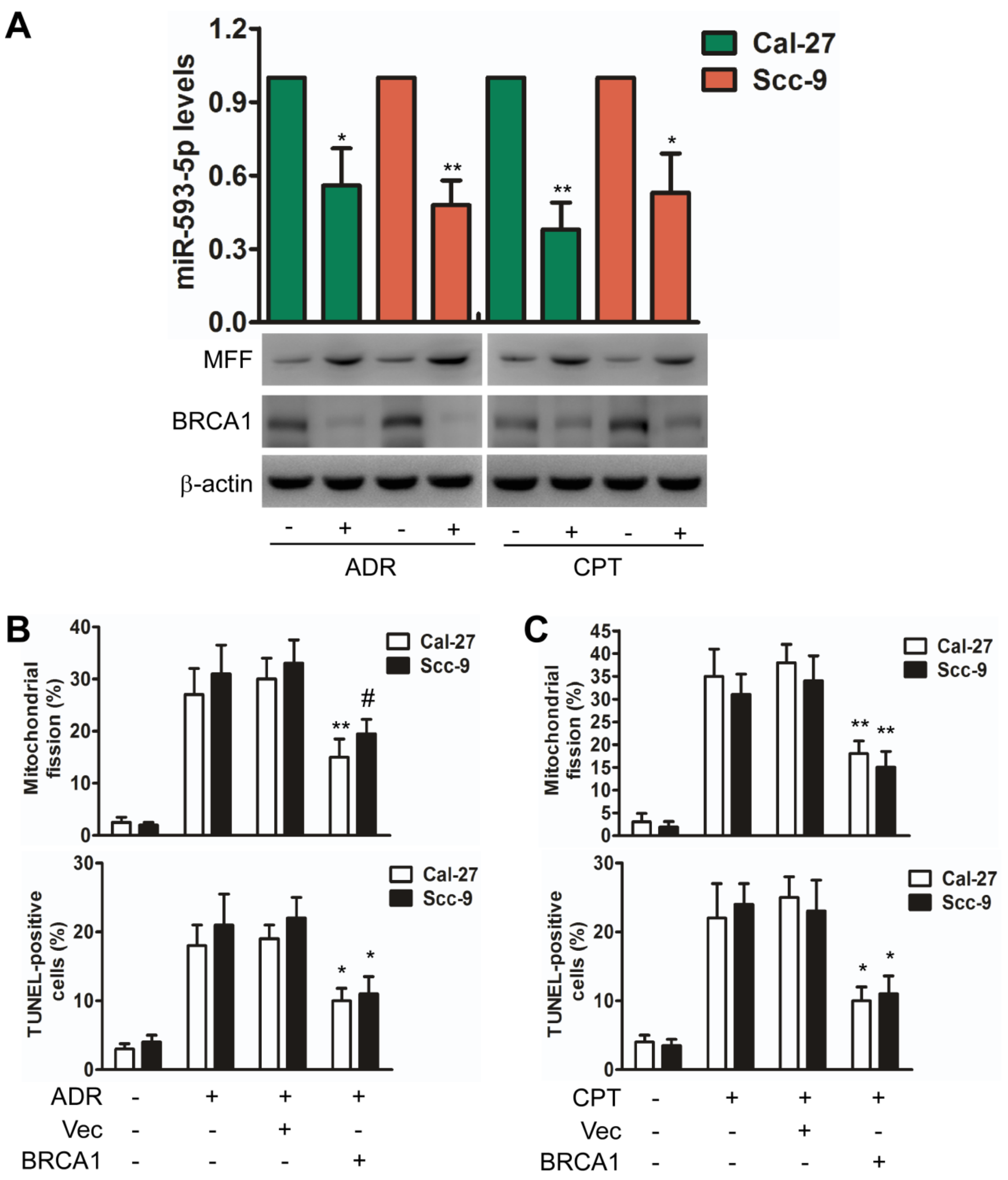

Supplementary Figure S7: The BRCA1–miR-593-5p–MFF axis participates in regulating mitochondrial fission and apoptosis upon treatment with adriamycin (ADR) and camptothecin(CPT) in TSCC cells. A, ADR and CPT induced mitochondrial fission with elevated MFF protein levels and decreased levels of miR-593-5p and BRCA1 protein. MFF and BRCA1 levels were analyzed via immunoblotting after ADR or CPT treatment, while miR-593-5p levels were detected using qRT-PCR. *P<0.01 versus no ADR or CPT treatment; **P<0.001 versus no

ADR or CPT treatment. B, BRCA1 attenuated mitochondrial fission and apoptosis in Cal-27 and Scc-9 cells under ADR treatment. Cal27 cells were transfected with BRCA1 expressing plasmids or empty vector (Vec). After 24 h of transfection, the cells were treated with ADR for 24 h. Apoptosis was detected using TUNEL. $^{\#}P<0.05$ versus ADR alone; $^{*}P<0.01$ versus ADR alone. $^{**}P<0.001$ versus ADR alone. C, BRCA1 attenuated mitochondrial fission and apoptosis in Cal-27 and Scc-9 cells under CPT treatment. Cal27 cells were transfected with BRCA1 expressing plismids or empty vector (Vec). After 24 h of transfection, the cells were treated with CPT for 24 h. Apoptosis was detected using TUNEL. $^{*}P<0.01$ versus CPT alone. $^{**}P<0.001$ versus CPT alone.

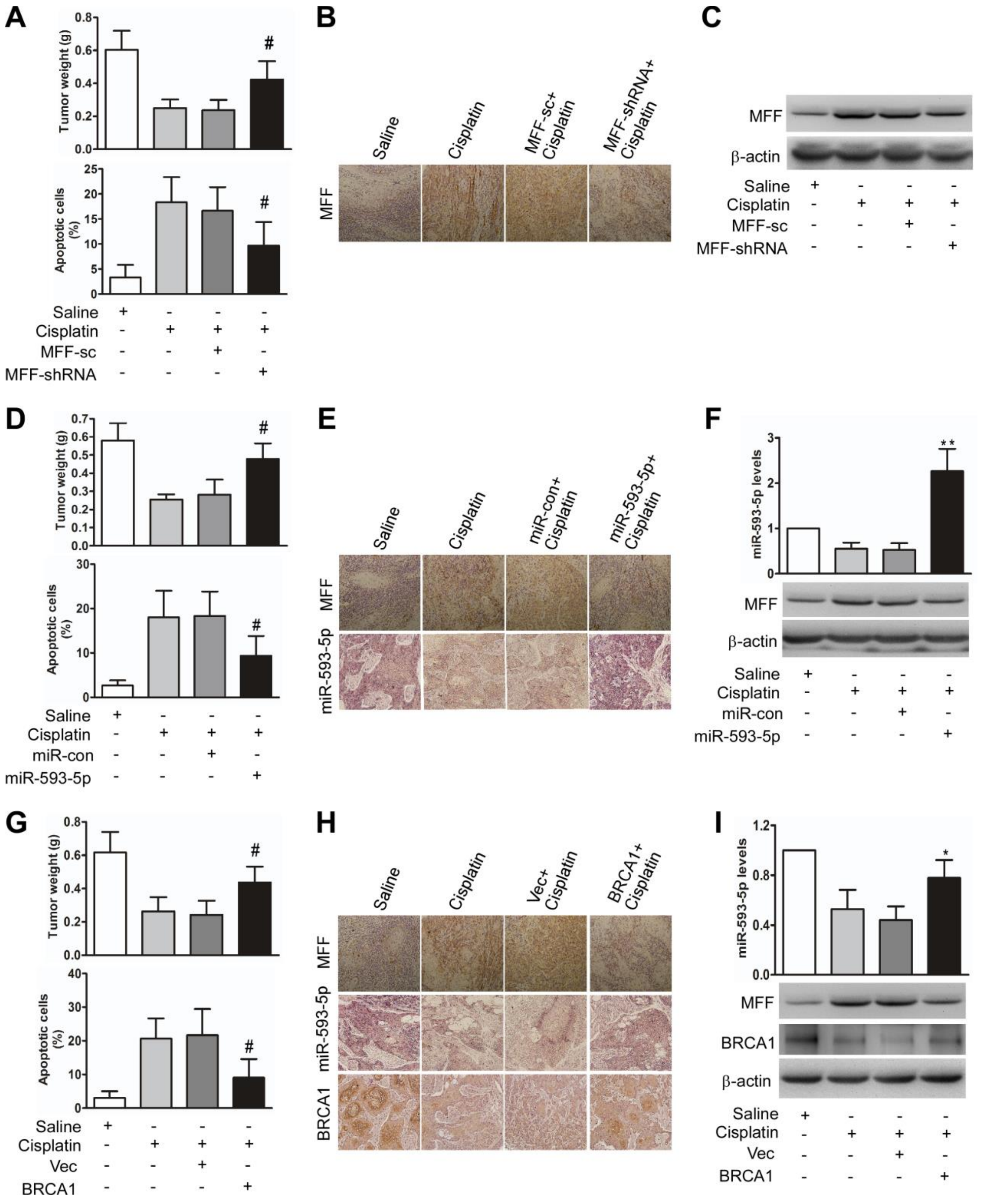

Supplementary Figure S8: BRCA1–miR-593-5p–MFF axis attenuates the cisplatin-induced inhibition of tumor growth in Cal-27 cell xenografts in BALB/c-nu mice. A, B, C, BALB/c-nu mice bearing Cal-27 cells with the stable expression of MFF shRNA or its scramble form (sc) were treated with saline or cisplatin. (A) Tumor weight and the apoptosis quantification of each group. The results are expressed as the means ± SEM. $^{\#}P< 0.05$ versus cisplatin alone. (B) MFF

expression of xenografts in each group was analyzed using immunohistochemistry (×200). n=24 slices from 6 xenograft tumors per group. (C) MFF expression of xenografts in each group was analyzed via immunoblotting. D, E, F, BALB/c-nu mice bearing Cal-27 cells with the stable expression of miR-593-5p or its control (con) were treated with saline or cisplatin. (D) Tumor weight and the apoptosis quantification of each group. The results are expressed as the means ± SEM. $^{\#}P < 0.05$ versus cisplatin alone. (E) MFF and miR-593-5p expression of xenografts in each group were analyzed via immunohistochemistry and in situ hybridization, respectively (×200). n=24 slices from 6 xenograft tumors per group. (F) MFF and miR-593-5p expression of xenografts in each group were analyzed via immunoblotting and qRT-PCR, respectively. The results are expressed as the means ± SEM. $^{**}P<0.001$ versus cisplatin alone. G, H, I, BALB/c-nu mice bearing Cal-27 cells with the stable expression of BRCA1 or empty vector (Vec) were treated with saline or cisplatin. (G) Tumor weight and the apoptosis quantification of each group. The results are expressed as the means ± SEM. $^{\#}P < 0.05$ versus cisplatin alone. (H) MFF, BRCA1 and miR-593-5p expression of xenografts in each group were analyzed via immunohistochemistry and in situ hybridization, respectively (×200). n=24 slices from 6 xenograft tumors per group. (I) MFF, BRCA1 and miR-593-5p expression of xenografts in each group were analyzed via immunoblotting and qRT-PCR, respectively. The results are expressed as the means ± SEM. $^{*}P < 0.01$ versus cisplatin alone.

Supplementary Table S1: Primers for each binding site in the ChIP–qPCR analysis; BS2-2 and BS2-1, as well as BS7-2 and BS7-1 share common primers because of close proximity. (We designed and applied two primer pairs for each biding site).

Biding sites	Sequences of the primers	
	Forward(5’-3’)	Reverse(5’-3’)
BS1	GAATGACAGTTTTCTCACCAGC	ACTGACTGGCACCCTTGG
	TGGCTGCTGTTCCTCAAG	CACAAGGTGACAGTGACTGACT
BS2	AGATGGATTTGACTCGGG	TGCCAAAATACATGTCTTCTAC
	TCAGATGGATTTGACTCG	TGCCAAAATACATGTCTTCT
BS3	GAGCATGTGGAGCAGATT	GCTGGCTAATTACAGCTG
	GAGCATGTGGAGCAGATT	GCTAATTACAGCTGCTGTTT
BS4	TGGGCAATGAAGAGGGAC	TGAGCAGGAACGATAGCAG
	GGCAATGAAGAGGGACAC	TGAGCAGGAACGATAGCAG
BS5	CAATGTAAAATTCTGGGG	ATGAAACCTCTGGAGTGG
	AGCTCAATGTAAAATTCTGG	ATGAAACCTCTGGAGTGG
BS6	GACCTTGGGTCAGTGGTC	CAGAGTCAGGGTGGGAGA
	CCTTGGGTCAGTGGTCCT	CAGAGTCAGGGTGGGAGA
BS7	CCTCCCTAACTTGGTCTAC	CCAGTGAGGCTCTGTAAG
	TCCCTAACTTGGTCTACTTT	CCAGTGAGGCTCTGTAAG
BS8	CCCAGGAACGGCTCTTGT	GAATGAACCGGGGCGGTG
	TGCCCAGGAACGGCTCTT	GAATGAACCGGGGCGGTG
NC	CCTTCGACCAGTCGGGTTTG	CTGTCCCGGAAAAGGCATCC

NC, negative control.
